# Enhancing Cellular Homeostasis: Targeted Botanical Compounds Boost Cellular Health Functions in Normal and Premature Aging Fibroblasts

**DOI:** 10.3390/biom14101310

**Published:** 2024-10-16

**Authors:** Ramona Hartinger, Khushboo Singh, Jesse Leverett, Karima Djabali

**Affiliations:** 1 Epigenetics of Aging, Department of Dermatology and Allergy, TUM School of Medicine, Munich Institute of Biomedical Engineering (MIBE), Technical University of Munich (TUM), 85748 Garching, Germany; ramona.hartinger@tum.de; 2 Amway Corporation, Innovation and Science, 7575 Fulton Street East, Ada, MI 49355, USA

**Keywords:** botanical compounds, cellular aging, JAK/STAT pathway, fibroblasts, skin, HGPS, premature aging, autophagy, inflammation, senescence

## Abstract

The human skin, the body’s largest organ, undergoes continuous renewal but is significantly impacted by aging, which impairs its function and leads to visible changes. This study aimed to identify botanical compounds that mimic the anti-aging effects of baricitinib, a known JAK1/2 inhibitor. Through in silico screening of a botanical compound library, 14 potential candidates were identified, and 7 were further analyzed for their effects on cellular aging. The compounds were tested on both normal aged fibroblasts and premature aging fibroblasts derived from patients with Hutchinson–Gilford Progeria Syndrome (HGPS). Results showed that these botanical compounds effectively inhibited the JAK/STAT pathway, reduced the levels of phosphorylated STAT1 and STAT3, and ameliorated phenotypic changes associated with cellular aging. Treatments improved cell proliferation, reduced senescence markers, and enhanced autophagy without inducing cytotoxicity. Compounds, such as Resveratrol, Bisdemethoxycurcumin, Pinosylvin, Methyl P-Hydroxycinnamate, cis-Pterostilbene, and (+)-Gallocatechin, demonstrated significant improvements in both control and HGPS fibroblasts. These findings suggest that these botanical compounds have the potential to mitigate age-related cellular alterations, offering promising strategies for anti-aging therapies, particularly for skin health. Further in vivo studies are warranted to validate these results and explore their therapeutic applications.

## 1. Introduction

The human skin, the body’s largest organ, entirely covers the external body surface, acting as a barrier against pathogens, environmental chemicals, and mechanical impacts [1,2,3]. As a multifunctional organ, it prevents dehydration and regulates temperature and sensory information [2]. The skin is organized into three layers: epidermis, dermis, and hypodermis [3]. The epidermis is the outermost layer, consisting of dynamically renewed epithelium [4]. It is composed of keratinocytes (arranged in multilayers), melanocytes, Langerhans cells, and Merkel cells [5,6]. Beneath the epidermis lies the dermis, separated by the basement membrane [7]. The dermis, the skin’s mesenchymal component, is scattered with a complex branched blood vessel [5,7]. It comprises connective tissue, including collagen and elastic fibers, along with lymphatic vessels, various fibroblast lineages, macrophages, nerve fibers, sweat glands, and hair follicles. This layer is instrumental in providing mechanical strength, nutrition supply, and circulatory functions [4,5,6]. Below the dermis lies the hypodermis, which is composed of loose connective tissue, adipose tissue, and various cell types, such as fibroblasts and adipocytes [6]. It serves as a protective layer against mechanical shocks and external temperatures (heat and cold) while also fulfilling the metabolic and energy storage roles [4].

The skin continuously undergoes renewal processes [4], yet aging, influenced by both internal and external factors, impairs its function and leads to visible changes such as wrinkles, dryness, thinning, altered pigmentation, and reduced barrier efficacy [8,9]. Aging is marked by a gradual deterioration in physiological functions and, in certain instances, cognitive abilities [10]. This complex process includes alterations at the cellular level, changes in metabolism, and modifications in the functionality and structure of tissues and organs, resulting in decreased self-regulation and impaired regenerative capacity [11].

Specifically in the skin, the age-related cellular limitations are critical: dermal cells show a decline in proliferative ability and enter a state known as replicative senescence [8,12]. This is followed by a reduced capacity of self-renewal within the skin [8,12]. Moreover, there is a reduction in melanocytes and Langerhans cells in the epidermal layer, leading to thinning of the epidermis and diminished barrier function [8,13]. The extracellular matrix within the dermis also undergoes significant changes during aging, affecting the skin’s mechanical strength, elasticity, and resilience [12,13]. Additional degenerative changes in the dermis include thinning, deterioration of the dermal–epidermal junctions, and degradation of key structural proteins like collagen and elastin [9,13,14]. The reduction in fibroblast numbers, which are crucial for the synthesis and structural organization of the extracellular matrix, along with an accumulation of senescent cells in both the epidermal and dermal layers impair cell proliferation and contribute to the aging skin phenotype [9,15,16]. Furthermore, the reduced interactions between fibroblasts and collagen fibers compromise the structural integrity and mechanical properties of aged skin [16,17,18].

The primary objective should be to maintain the functionality of fibroblasts and enhance their resilience to the aging process. Previous studies have shown that premature senescence and inflammation significantly contribute to the aging of fibroblasts [19,20]. Specifically, the JAK1/2-STAT1/3 signaling pathway is notably overactivated during cellular aging. Utilizing baricitinib (bar), a JAK1/2 inhibitor that is approved by the Food and Drug Administration (FDA) [21], demonstrated promising results in reducing senescence levels and proinflammatory markers, thereby improving cellular homeostasis and mitigating age-related cellular alterations [19,20]. Building on these insights, we conducted an in silico screening of botanical compound libraries to identify molecules that mimic the anti-aging effects of baricitinib. This screening process led to the identification of 14 potential candidates. These compounds were subsequently tested in vitro to evaluate their ability to inhibit the JAK/STAT pathway and to assess their effectiveness in ameliorating the phenotypic changes linked to cellular aging in both normal aged fibroblasts and premature aging fibroblasts derived from patients with Hutchinson–Gilford progeria syndrome (HGPS, OMIM 176670) [22].

HGPS is a rare genetic disorder marked by premature aging, resulting from a de novo autosomal dominant mutation in the LMNA gene, which encodes Lamin A/C [23,24]. Specifically, the point mutation c.1824C>T, p.G608G, activates a cryptic splice side, resulting in the loss of 50 amino acids and the accumulation of a truncated Lamin A protein, identified as progerin [25,26,27]. Patients exhibit symptoms similar to those of physiological aging, including hair loss, atherosclerosis, lipodystrophy, sclerotic skin, reduced bone mineral density, joint contractures, and abnormal growth patterns [22,25,27]. Symptoms can manifest as early as six months of age and progress rapidly thereafter [25,28], with an average life expectancy of only about 14.6 years [29]. Due to its severe premature aging effects, HGPS serves as an excellent model for studying accelerated aging at the cellular level, where changes include premature senescence, altered gene expression, DNA damage, and mitochondrial dysfunction [19,20,30,31].

In the present research, HGPS fibroblasts were employed to assess the effectiveness of botanical compounds in treating aging-related cellular phenotypic changes. From the initial 14 botanical compounds identified, 7 were selected for further analysis in both control and HGPS fibroblasts. This analysis focused on assessing their impact on cell proliferation, cellular aging phenotype, and cytotoxicity. The results demonstrated that these seven botanical compounds effectively inhibit the JAK/STAT pathway and ameliorate the effects associated with replicative senescence and cellular homeostasis in both control and HGPS fibroblasts.

## 2. Materials and Methods

### 2.1. Virtual Screening

#### 2.1.1. Protein and Ligand Preparation

The protein structures (PDB IDs: 6VN8 and 6HZU) were set up for docking with the use of the “Protein preparation wizard” tool in Schrödinger suite [32]. The protocol involved removing water molecules and cofactors, correcting any misidentified elements, adding hydrogen atoms, determining bond orders, and optimizing hydrogen bonds. The assignment of hydrogen bonds was performed using PROPK at a pH of 7.0, which also included optimizing the orientation of hydroxyl groups, the side-chain amide groups of Asn and Gln, and the charge states of His residues. Protein structures were then minimized to a root-mean-square deviation (RMSD) limit of 0.3 Å from the initial structure, employing the OPLS3 force field [33]. The proteins that were prepared were then used for grid generation with the “Receptor Grid Generation” panel in the Glide module of the Schrödinger suite [34]. The binding site was determined by selecting key residues at the subsites. The phytochemicals were prepared using LigPrep3.4 with Epik3.2, set at a pH of 7.0.

The phytochemical library was generated using the Maestro LigPrep tool from Schrödinger [35], which applied the OPLS4 force field, optimized the structures, and added hydrogen atoms. Epik was also employed to assign likely protonation states within a pH range of 7 ± 2 and to determine tautomeric forms for each compound.

#### 2.1.2. Molecular Docking

Virtual screening was conducted using the Glide program3 (version 2024-1), with ligand-flexible docking of the prepared ligands to the protein binding sites carried out at two levels: standard precision (SP) and extra precision (XP). This process utilized the Virtual Screening Workflow protocol of GLIDE.

### 2.2. Cell Culture and Compound Treatment

The study utilized the following human primary dermal fibroblast cell lines: Control cell strains—GM05757C (7-year-old male), GM05567A (12-year-old male), HGFDFN369 (33-year-old male), HGMDFN368 (31-year-old female), and GM01651C (13-year-old female) without mutations; HGPS cell strains—HGADFN003 (2-year-old male), HGADFN164 (4-year-old female), HGADFN271 (1-year-old male), and HGADFN127 (3-year-old female) with mutation on LMNAExon 11, heterozygous c.1824C>T (p.Gly608Gly). Human control primary dermal fibroblast cells were sourced from the Coriell Institute for Medical Research (Camden, NJ, USA), while HGPS cells were acquired from the Progeria Research Foundation Cell and Tissue Bank.

The primary fibroblast cultures were maintained in a cell incubator (Binder, Tuttlingen, Germany, 9140-0046) in DMEM (Thermo Fisher—Gibco, Waltham, MA, USA, D6429) supplemented with 15% fetal bovine serum (FBS; Thermo Fisher—Gibco, Waltham, MA, USA, 10270106), 1% L-glutamine (Thermo Fisher—Gibco, Waltham, MA, USA, 25030081), 1% gentamycin (Thermo Fisher—Gibco, Waltham, MA, USA, 15710049), and 1% penicillin/streptomycin (Thermo Fisher—Gibco, Waltham, MA, USA, 1514022) under a 5% CO_2_ atmosphere and 37 °C. Fibroblast monocultures were subcultured and used at 80% confluence. The senescence level of the fibroblast cultures was between 10 and 15%, and the passage numbers are specified in Table 1.

For the botanical compound treatment, dermal fibroblasts were cultured, and the medium was mock (no compound) or supplemented with the botanical compound in defined concentration.

### 2.3. Senescence-Associated β-Galactosidase Assay

The β-Galactosidase Assay was implemented to determine the cellular senescence level of the fibroblast monocultures, as formerly described by Dimri et al. (1995) [36]. The fibroblasts were first washed with phosphate-buffered saline (PBS) (Sigma-Aldrich, St. Louis, MO, USA) and then fixed using 2% formaldehyde (Sigma-Aldrich, St. Louis, MO, USA, 104003) and 0.2% glutaraldehyde (Sigma-Aldrich, St. Louis, MO, USA, G5882) for 5 min. Following this, the cells were washed twice with PBS and incubated with SA-β-Gal staining solution overnight at 37 °C (without CO_2_). This solution consisted of 2 mM MgCl_2_ (Sigma-Aldrich, M-1028), 5 mM potassium ferrocyanide (Sigma-Aldrich, P9387), 5 mM potassium ferricyanide (Merck KGaA, 104973, Darmstadt, Germany), 150 mM NaCl (Sigma-Aldrich, St. Louis, MO, USA, 310166), 0.5 mg/mL 5-bromo-4-chloro-3-indolyl β-D-galactoside (X-gal) (Sigma-Aldrich, St. Louis, MO, USA, 3117073001), and 40 mM citrate/sodium phosphate buffer (pH 6.0). On average, 1000 cells per sample were counted, and blue-stained cells were identified as senescent.

### 2.4. Determination of Cell Number and Cytotoxicity

To determine the cell number and viability, cells were seeded in a density of 3.5 × 10^3^ cells in a 24-well plate with or without treatment. The fibroblasts were trypsinized using trypsin-EDTA (Thermo Fisher—Gibco, Waltham, MA, USA, 25200056) and analyzed with the Muse^TM^ Cell Analyzer (Merck Millipore, Burlington, MA, USA) using DNA-binding dyes to assess the fluorescent signal.

For cytotoxicity, the percentage of dead cells was calculated using the following formula:

% *dead cells* = 100% − % *viable cells.*

Cumulative population doubling (CPD) was determined using the following formula:

*n* = 3.32 (*log cells harvested* − *log cells seeded*) + *x*,

where *n* is the CPD number at this cell culturing state, and *x* = the former CPD [37].

### 2.5. Cell Cycle Analysis

The percentage of cells in the G0/G1, S, and G2/M phases of the cell cycle was determined using the Muse Cell Cycle Assay kit (Cytec Biosciences, Amsterdam, The Netherlands). This kit includes a premixed reagent containing the nuclear DNA intercalating stain propidium iodide (PI) and RNAse A. The different phases of the cell cycle were identified based on PI staining of DNA content. The quantification of the signal was performed with the Muse™ Cell Analyzer (Merck Millipore, Burlington, MA, USA).

### 2.6. Western Blot

Cells were washed once with PBS and collected by scraping on ice with lysis buffer (150 mM NaCl, 1% Triton, 1% SDS, 1 mM EDTA, 50 mM Tris, 45% 2xLämmli sample buffer (BioRad, Hercules, CA, USA, 1610737), 3% B-Mercaptoethanol (BioRad, 1610710), 0.5% Protease Inhibitor (Thermo Fisher, 78430), 0.5% Phosphatase Inhibitor (Thermo Fisher, 78426), and 200 mM PMSF). Total protein concentration was measured using the Bradford assay, with BSA (BioRad Laboratories, 5000206, Hercules, CA, USA) serving as the standard. An amount of 20 μg protein lysate was separated in an 8% or 15% gel with electrophoresis and then transferred onto nitrocellulose membranes using the wet-transfer method. The membranes were blocked with 5% non-fat milk for 1 h at RT and incubated with the following primary antibodies: anti-lamin A/C (E1, sc-376248, Santa Cruz Biotechnology, Dallas, Texas, USA, 1:10,000), P-STAT1 (58D6, Cell Signaling, Leiden, The Netherlands, 1:1000), STAT1 (D1K9Y, Cell Signaling, 1:1000), P-STAT3 (D3A7, Cell Signaling, 1:1000), STAT3 (124H6, Cell Signaling, 1:1000), p62 (SQSTM1, 66184-1-Ig, Proteintech, Manchester, UK, 1:1000), p16 (INK4A, JC8, sc-56330, Santa Cruz, 1:1000), p21 (Wafa1/Cip 1, DCS60, Cell Signaling, 1:1000), LC3B (LC3A/B, D3U4C, 12741, Cell Signaling, 1:2000), P-AMPK (Phospho-AMPKα, Thr172, 40H9, Cell Signaling, 1:1000), AMPK (AMPKα, Cell Signaling, 1:1000), P-NfκB (Phospho-NF-κB p65, Ser536, 93H1, Cell signaling, 1:2000), NfκB (NF-κB p65, D14E12, Cell Signaling, 1:2000), and anti-GAPDH (G9545, Sigma-Aldrich, 1:5000) overnight at 4 °C. Thereupon, the membrane was washed three times with TBS-Tween and incubated for 1 h at RT with horseradish peroxidase-conjugated anti-rabbit (1:5000) or anti-mouse (1:5000) secondary antibodies (Jackson ImmunoResearch Laboratories, West Grove, Pennsylvania, USA). After washing with TBS-Tween, luminol-enhanced chemiluminescence was conducted, and the signals were visualized with the ChemiDoc™ MP. Quantification was achieved using ImageJ software (Version 1.5, NIH, Bethesda, MD, USA), and the blots were normalized to GAPDH expression levels (internal control).

### 2.7. Immunocytochemistry

Fibroblasts were cultured on glass cover slips and fixed for 10 min with 2% PFA (Merck KGaA, 104005). After three washes, each 5 min with PBS, the cells were permeabilized for 5 min with 0.2% Triton X-100 in PBS and then washed with PBS for 5 min. Following permeabilization, the fibroblasts were blocked for 30 min at RT with 10% FBS (Thermo Fisher—Gibco, 10270106) in PBS and incubated at 4 °C overnight with the following primary antibodies: rabbit anti-γ-H2AX (2577, Cell Signaling, 1:1000), mouse anti-Lamin B1 (Santa Cruz Biotechnology, 1:200), rabbit anti-Vimentin (ab137321, Abcam, Cambridge, UK, 1:1200), and mouse anti-p21 (Wafa1/Cip 1, DCS60, Cell Signaling, 1:600). The following day, cells were washed four times with blocking buffer and then incubated for 1 h at RT with the following secondary antibodies: affinity-purified Alexa Fluor^®^ 488 or 555 conjugated anti-mouse/-rabbit antibodies (Life Technologies, Thermo Fisher, Waltham, MA, USA, A21202 anti-mouse-488 and A31572 anti-rabbit-555, 1:1000). After washing two times with blocking buffer and twice with PBS, fibroblasts were counterstained with DAPI Vectashield mounting medium (Vector Laboratories, Burlingame, CA, USA, VEC-H-1200), and images were taken using a Keyence BZ-X810 fluorescence microscope (KEYENCE DEUTSCHLAND GmbH, Neu-Isenburg, Germany).

### 2.8. Measurement of ROS

Reactive oxygen species (ROS) in live fibroblasts were quantified using a DCFDA—Cellular ROS Assay Kit/Reactive Oxygen Species Assay Kit (ab113851, Abcam, Germany). 2′,7′-dichlorofluorescin diacetate (DCFDA) is a cell-permeant reagent that undergoes deacetylation and transforms into a non-fluorescent substance in living cells. ROS oxidize it into 2′,7′-dichlorofluorescein (DCF), an intensely fluorescent compound.

Fibroblasts with and without treatment were seeded in triplicates in 96-well plates (2.5 × 10^4^ cells/well) and allowed to attach overnight. After a single wash with assay buffer, the cells were stained for 30 min at 37 °C with 25 μM DCFDA and then washed once more with assay buffer. Fluorescence was assessed using a FLUOstar Omega Microplate Reader (BMG Labtech, Ortenberg, Germany) with excitation and emission wavelengths set to 485 nm and 520 nm, respectively. All measurements were conducted in three technical replicates for each cell strain and treatment condition.

### 2.9. Autophagy Activity

Fibroblast monocultures with and without treatment were seeded in triplicates in 96-well plates at a density of 5 × 10^4^ cells/well and allowed to attach overnight. Autophagy activity and the influence of different botanical compound treatments were analyzed using Cayman’s Autophagy/Cytotoxicity Dual Staining Kit (Cayman Chemical Company, Ann Arbor, Michigan, USA). Autophagic vacuoles in cells were stained and detected with monodansylcadaverine (MDC), an autofluorescent compound that incorporates into multilamellar bodies. Adherent cells were stained with MDC (1:100) at 37 °C for 10 min and washed once with assay buffer. The intensities of the autophagic vacuoles were measured using a FLUOstar Omega Microplate Reader (BMG Labtech, Germany) with an excitation wavelength of 355 nm and an emission wavelength of 520 nm. All measurements were performed in three technical replicates per cell strain and treatment condition.

### 2.10. Gene Expression Analysis

Cell pellets were prepared out of fibroblast cultures by trypsinization, and RNA was isolated with the GeneJET RNA Purification Kit (Thermo Fisher). The RNA amount was measured using a NanoDrop spectrophotometer (NanoDrop ND-1000, Thermo Fisher). cDNA was prepared by reverse transcribing 500 ng of RNA with the High-Capacity cDNA Reverse Transcription Kit (Thermo Fisher). Real-time PCR primers were designed using NCBI/Primer-BLAST [38] and obtained from Thermo Fisher. The quantified genes and their primers can be found in the primer list (Table S1). The real-time PCR was conducted on a StepOnePlus™ Real-Time PCR System (Thermo Fisher) with the PowerUp™ SYBR™ Green Master Mix (Applied Biosystems™, Thermo Fisher) by using 300 nM of each primer and 50 ng of the template in a total reaction volume of 20 µL. The thermal cycling program started with an initial denaturation at 95 °C for 20 s, followed by 45 cycles of denaturation at 95 °C for each 3 s and annealing/extension at 60 °C for 30 s. Amplification signals were detected between cycles 10 and 40. GAPDH served as the endogenous control, and every experiment was performed in triplicates with three biological replicates.

### 2.11. Statistical Evaluation and Graphics

For each cell strain, three biological replicates were cultured and analyzed under the different treatment conditions. An amount of 1000 cells was counted for senescence and immunocytochemistry.

The findings are shown as average value plus or minus the standard deviation (mean ± SD) and were obtained by using the student’s *t*-test, one-way anova, and two-way anova to assess the variation between two distinct groups. Calculations and graphs were produced using GraphPad Prism (Version 6.01, GraphPad, La Jolla, California, USA). Statistical significance is indicated by the following symbols: ns, not significant (*p* > 0.05); * *p* ≤ 0.05; ** *p* ≤ 0.01; *** *p* ≤ 0.001; and **** *p* ≤ 0.0001.

## 3. Results

### 3.1. In Silico Analysis

Baricitinib, a known inhibitor of JAK1 and JAK2 signaling, has been previously reported to ameliorate several hallmarks of cellular aging in both normal and Hutchinson–Gilford Progeria Syndrome (HGPS) cells [19,20]. Prompted by these findings, we initiated an in silico screening to identify botanical compounds that could interact with JAK1 and JAK2 in a manner similar to baricitinib.

In this screening, molecular docking techniques were employed to assess the binding affinity and orientation of various botanical compounds at the active sites of JAK1 and JAK2. We used the CMAUP database [39], which includes approximately 48,000 phytochemicals, targeting the ATP-binding site of Type I JAK-1/JAK-2 inhibitors, like baricitinib, ruxolitinib, and fedratinib, all previously reported to counteract aging hallmarks in both normal and Hutchinson–Gilford Progeria Syndrome (HGPS) cells [19,20].

**Figure 1:**
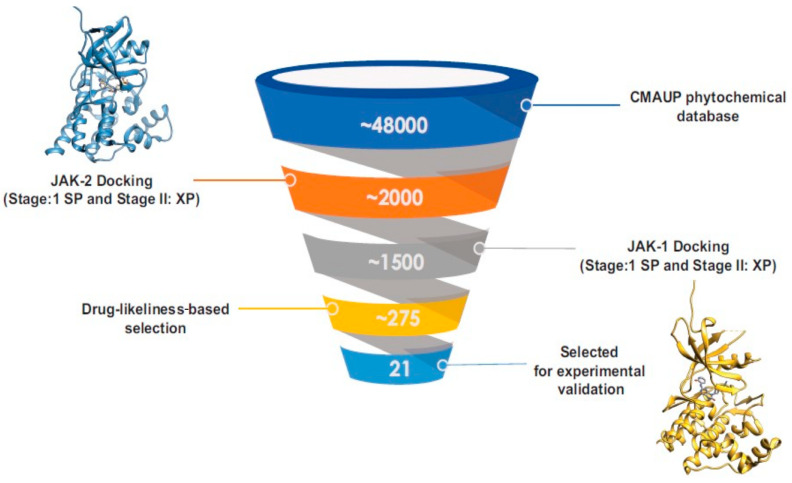
The screening workflow used in virtual screening. Out of approximately 48,000 phytochemicals in the database, 2000 molecules showed a docking score above −6 kcal/mol for JAK-2. Among these, about 1500 molecules exhibited docking scores above −6 kcal/mol for JAK-1. Following this initial screening, approximately 275 phytochemicals were selected based on drug-likeness criteria (Lipinski rule of 5). From this subset, 21 molecules were prioritized for experimental validation.

Figure 1 illustrates the screening process used to prioritize the best-performing molecules. The study aimed to identify non-specific inhibitors, so the molecules with a docking score above −6.0 kcal/mol for JAK-2 were also investigated for their binding ability to JAK-1. Based on the selection criteria of drug-likeliness, 21 molecules that exhibited high docking scores for both JAK-1 and JAK-2 were selected for further study. This computational approach facilitated the prediction of interaction patterns and the identification of compounds with potential inhibitory effects similar to those of baricitinib, thus indicating candidates for subsequent experimental validation (Table 2). Table 2 presents the structures, docking scores, and the chemical properties of the selected compounds. Supplementary Table S1 lists all 275 molecules that were selected based on the XP docking scores and drug-likeliness.

### 3.2. Botanical Compound Concentration Determination

To determine if an in silico-identified botanical compound can inhibit JAK1 and JAK2 signaling, control cells with a senescence of approximately 15% were cultured, and different compound concentrations were analyzed in a 3-day short-term treatment. The activation of JAK1/JAK2 signaling can be detected via Western blot analysis of phosphorylated STAT1 (P-STAT1) and STAT3 (P-STAT3), which serve as direct readouts for the pathway activity [40,41]. The compound efficiency was measured by analyzing the amount of P-STAT1 and P-STAT3 protein levels in cells treated with the various compounds. In addition, the appearance and growth of the cells were examined daily.

**Figure 2:**
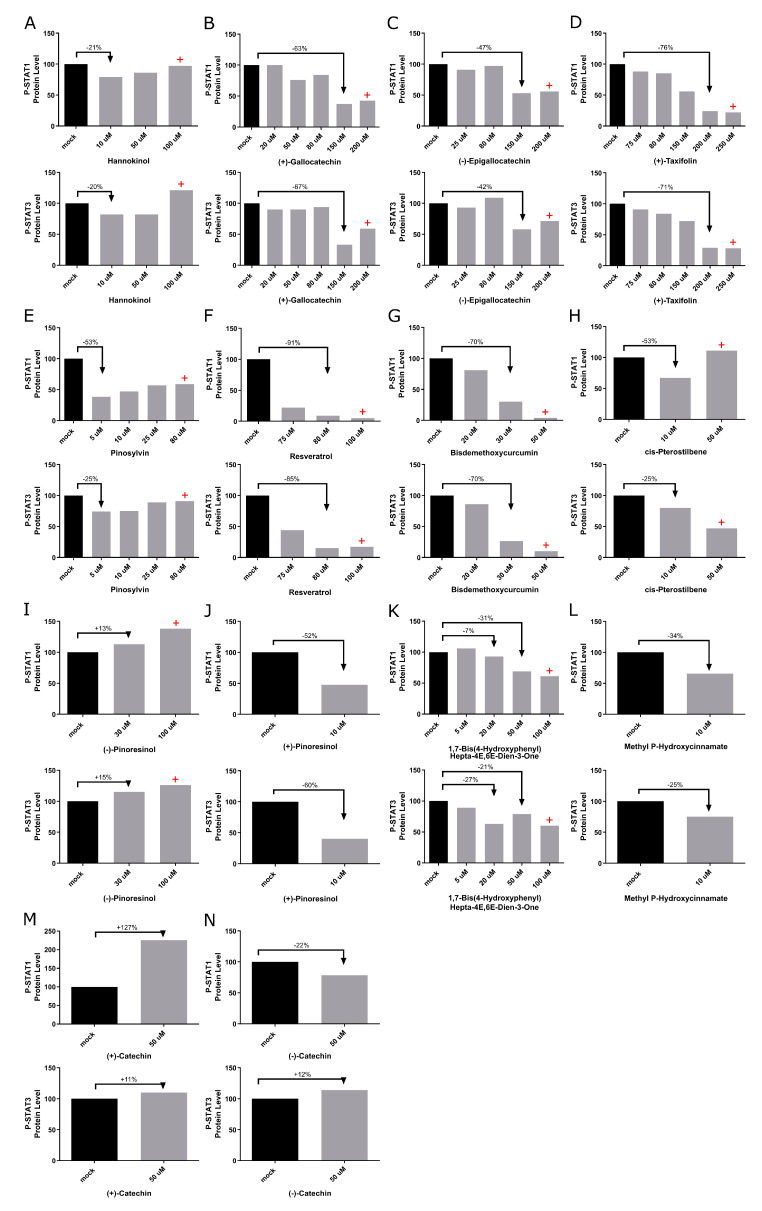
Detection of JAK-STAT inhibition by candidate botanical compounds. (**A**–**N**) P-STAT protein levels in control fibroblasts (5757C, SNS 15%) were quantified after treatment with specified botanical compounds for 3 days. Western blots are shown in Figure S1. The levels of P-STAT1 and P-STAT3 were quantified using Western blot analyses and normalized to GAPDH. Bars marked with a red cross (+) indicate concentrations at which the compounds exhibited cytotoxic effect, as evidenced by increased cell death compared to mock-treated counterparts.

Control fibroblasts treated with the botanical compounds Hannokinol, (+)-Gallocatechin, (−)-Epigallocatechin, (+)-Taxifolin, Pinosylvin, Resveratrol, Bisdemethoxycurcumin, cis-Pterostilbene, (+)-Pinoresinol, 1,7-Bis-(4-Hydroxyphenyl)Hepta-4E,6E-Dien-3-One, and Methyl P-Hydroxycinnamate showed decreased levels of P-STAT1 and P-STAT3 levels at day 3 (Figure 2). When toxicity was observed, a red cross (+) was added to the graph at the indicated concentration. All Western blot analyses are shown in Figure S1. Hannokinol exhibited moderate inhibition of P-STAT1 and P-STAT3, reducing their activity by approximately 20%. In contrast, compounds, such as (+)-Gallocatechin, (−)-Epigallocatechin, (+)-Taxifolin, Pinosylvin, Resveratrol, Bisdemethoxycurcumin, cis-Pterostilbene, (+)-Pinoresinol, and Methyl P-Hydroxycinnamate, demonstrated significantly stronger inhibitory effects, with a reduction of P-STAT1 and P-STAT3 activity ranging from 25% to 91% (Figure 2A–H,J–L). High-dose treatments with these compounds (Hannokinol, (+)-Gallocatechin, (−)-Epigallocatechin, (+)-Taxifolin, Pinosylvin, Resveratrol, Bisdemethoxycurcumin, cis-Pterostilbene, and 1,7-Bis-(4-Hydroxyphenyl)Hepta-4E,6E-Dien-3-One), indicated by a red cross on the graphs, led to a notable decrease in cell growth or increase in cell death when compared to the mock-treated cells. This cytotoxic effect was systematically observed and recorded during daily monitoring of the cell cultures (Figure 2). Treatment with (−)-Catechin resulted in a 12% increase in P-STAT3 protein levels compared to the mock treatment (Figure 2N). Similarly, treatment with (−)-Pinoresinol and (+)-Catechin led to elevated levels of P-STAT1 and P-STAT3 levels, with increases ranging from 11% to 27% (Figure 2I,M).

Overall, 12 botanical compounds identified through in silico analysis effectively decreased the levels of P-STAT1 and P-STAT3. Specifically, 9 of these compounds achieved reductions exceeding 25%. Consequently, the effects of (+)-Gallocatechin, (−)-Epigallocatechin, (+)-Taxifolin, Pinosylvin, Resveratrol, Bisdemethoxycurcumin, cis-Pterostilbene, (+)-Pinoresinol, and Methyl P-Hydroxycinnamate were further analyzed using long-term treatment studies.

### 3.3. Long-Term Treatment Screening

To investigate the potential beneficial and adverse effects of these nine preselected botanical compounds in long-term treatments, it is essential to understand the initial cellular response. Upon first exposure, cells may exhibit improved functionality and survival, as observed at day 3 and often considered an adaptive response to mild stress [42,43]. However, prolonged exposure can lead to intracellular accumulation of the compounds, potentially overwhelming the cell’s adaptive mechanisms and resulting in toxicity. This accumulation can trigger a shift towards apoptosis, thereby increasing the rate of cell death [44]. To examine these dynamics over an extended period, we conducted a long-term treatment study lasting 7 days. During this period, we monitored cell proliferation using cumulative population doubling (CPD) and assessed cytotoxicity to quantify the effects of each compound.

**Figure 3:**
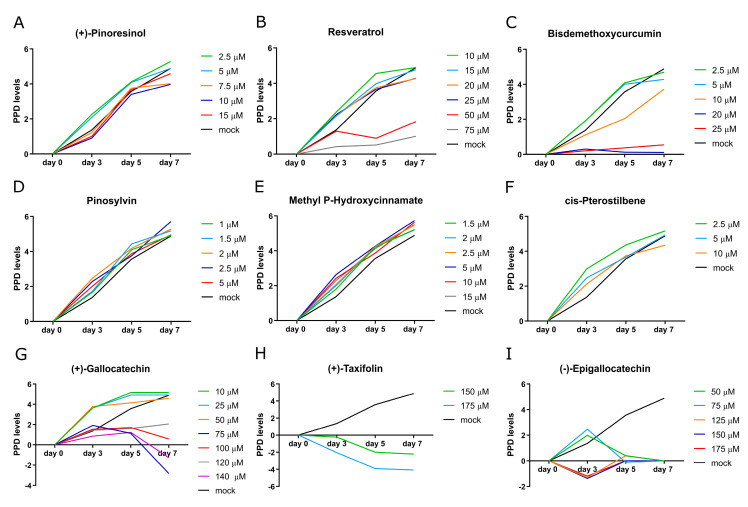
Cumulative population doubling (CPD) was measured after 7 days of long-term treatment with botanical compounds at indicated various different concentrations in control fibroblasts (representative strain 5757C, SNS~15%). Mock-treated control cells are depicted in black.

Compared to mock-treated control fibroblasts, treatments with Pinosylvin, Methyl P-Hydroxycinnamate, and cis-Pterostilbene showed an increase in cell growth across all tested concentrations. Additionally, an improvement in cell proliferation was observed with lower concentrations of (+)-Pinoresinol, Resveratrol, Bisdemethocycurcumin, and (+)-Gallocatechin (Figure 3A–C,G). However, higher concentrations of these compounds led to reduced cell growth; (+)-Gallocatechin showed cytotoxic effects at concentrations of 100 µM, 120 µM, and 140 µM (Figure 3G). Conversely, a 7-day treatment with (+)-Taxifolin and (−)-Epigallocatechin negatively impacted cell growth and exhibited cytotoxicity (Figure 3H–I), leading to their exclusion from further investigation.

Collectively, these findings indicate that seven botanical compounds ameliorated the proliferation of aged control fibroblasts and reduced the protein levels of P-STAT1 and P-STAT3 without inducing cytotoxicity, thereby justifying their selection at working concentrations for further studies (Table 3).

### 3.4. Treatment with Botanical Compounds Improved the Proliferative Rate in Both Control and HGPS Cells and Delayed the Onset of Senescence

**Figure 4:**
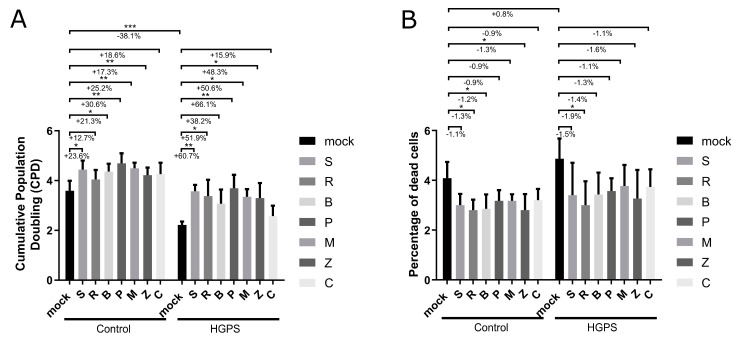
Cumulative population doubling (CPD) and percentage of dead cells in control and HGPS fibroblasts. Control fibroblast (strains 5757C, 5567A, F369, M368) and HGPS fibroblast (strains P003, P127, P271) cultures with a senescence level of ~15% were grown and treated under varying conditions for 7 days. The treatment groups included no compound (mock), 2.5 μM (+)-Pinoresinol (S), 10 μM Resveratrol (R), 2 μM Bisdemethoxycurcumin (B), 1 μM Pinosylvin (P), 1.5 μM Methyl P-Hydroxycinnamate (M), 2.5 μM cis-Pterostilbene (Z), and 10 μM (+)-Gallocatechin (C). (**A**) Population doubling determined on day 7 of cultivation. (**B**) Percentage of dead cells on day 7 of cultivation. (**A**,**B**) Values are presented as mean ± SD (n = 4 for control, n = 3 for HGPS); * *p* < 0.05; ** *p* < 0.01; *** *p* < 0.001; assessed using unpaired *t*-test and one-way anova.

To determine whether selected botanical compounds could improve age-related malfunctions in control and HGPS cells, a seven-day treatment regimen was implemented. Cells were treated either with no compound (mock) or with specific concentrations of the following botanical compounds: 2.5 μM (+)-Pinoresinol, 10 μM Resveratrol, 2 μM Bisdemethoxycurcumin, 1 μM Pinosylvin, 1.5 μM Methyl P-Hydroxycinnamate, 2.5 μM cis-Pterostilbene, and 10 μM (+)-Gallocatechin. Each compound was applied separately. Cell proliferation, measured by the cumulative population doubling (CPD), and cytotoxicity were quantified on day 7 to determine the effect of these treatments (Figure 4).

We initially evaluated the impact of the botanical compounds on normal human fibroblasts (controls) and observed positive effects. To further explore their potential, we extended our testing to a model of premature cellular aging, using Hutchinson–Gilford Progeria Syndrome (HGPS) cells [45]. This approach enabled us to assess the efficacy of the compounds in a system characterized by accelerated aging. Compared to mock treatments, both control and HGPS fibroblast cultures exhibited improved cell growth when treated with the botanical compounds (Figure 4). There was a significant increase in CPD in control groups treated with (+)-Pinoresinol, Bisdemethoxycurcumin, Pinosylvin, Methyl P-Hydroxycinnamate, and cis-Pterostilbene. As expected, the mock-treated HGPS fibroblasts showed growth retardation relative to the mock-treated control fibroblasts (Figure 4A). However, the growth rate of HGPS cells significantly improved when treated with (+)-Pinoresinol, Resveratrol, Pinosylvin, Methyl P-Hydroxycinnamate, and cis-Pterostilbene (Figure 4A).

Furthermore, all compound treatments showed no cytotoxic effects at the selected concentrations (Figure 4B). The percentage of dead cells was lower compared to mock treatments in control cells treated with Bisdemethoxycurcumin or cis-Pterostilbene (Figure 4B). A similar reduction in cell death was observed in HGPS cultures treated with Resveratrol (Figure 4B). The cell toxicity observed in the mock-treated control cultures reflects the baseline level of cell death inherent to the experimental procedures (Figure 4B).

**Figure 5:**
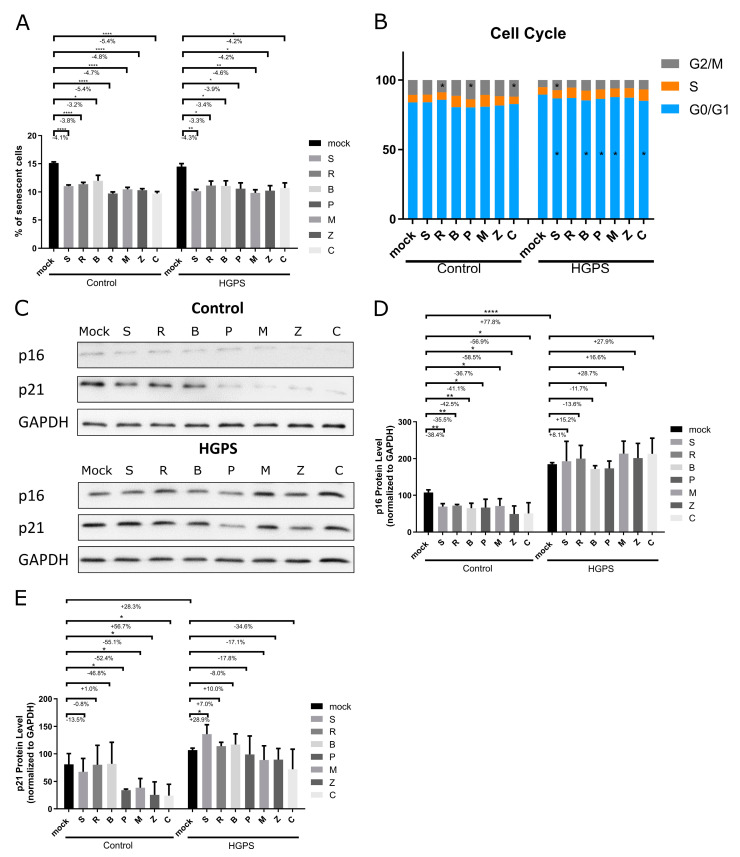
Replicative senescence levels and cell cycle profiles of control and HGPS fibroblasts under different compound treatment conditions. Control fibroblasts (strains 5757C, 5567A, F369, M368) and HGPS fibroblasts (strains P003, P127, P271) with a senescence level of ~15% were cultured and treated for 7 days with the following: no compound (mock), 2.5 μM (+)-Pinoresinol (S), 10 μM Resveratrol (R), 2 μM Bisdemethoxycurcumin (B), 1 μM Pinosylvin (P), 1.5 μM Methyl P-Hydroxycinnamate (M), 2.5 μM cis-Pterostilbene (Z), and 10 μM (+)-Gallocatechin (C). (**A**) Percentage of senescent cells in control and HGPS groups (Control: n = 4, HGPS: n = 3). (**B**) Cell cycle profiles of control and HGPS groups. Relative percentages of cells in the G0/G1, S, and G2/M phases are shown. DNA was stained with propidium iodine (PI) (Control: n = 4, HGPS: n = 3). (**C**) Representative images of Western blot analyses for p16 and p21 proteins in total protein extracts. Normalized to GAPDH. Original western blots can be found at Figure S3. (**D**,**E**) Quantification of p16 and p21 protein levels normalized to GAPDH (**E**). Graphs display means ± SD (Control and HGPS n = 3); * *p* < 0.05; ** *p* < 0.01; **** *p* < 0.0001; assessed using unpaired *t*-test and one-way anova.

Following the assessment of growth and cytotoxicity, we further investigated the effects of these botanical compounds on cellular senescence and the cell cycle in both control and HGPS cultures, which had a baseline senescence (SNS) level of ~15%. These cells were treated with the indicated compounds and analyzed on day 7 (Figure 5A,B).

Senescence levels, determined by senescence-associated β-Galactosidase (β-Gal) staining, were significantly reduced in both control and HGPS fibroblasts across all treatment conditions compared to their mock-treated counterparts (Figure 5A). Specifically, in control cells, Pinosylvin and (+)-Gallocatechin significantly decreased the senescence levels by 5.4%. In HGPS cells, Methyl P-Hydroxycinnamate exhibited the most pronounced effect, reducing senescence by 4.6% (Figure 5A).

The observed delay in senescence and improved growth rates were corroborated by the cell cycle profiles from the same compound treatments. Cell cycle analyses indicated a higher proportion of HGPS cells in the S and G2/M phases across all treatments (Figure 5B). Control fibroblasts treated with Bisdemethoxycurcumin, Pinosylvin, Methyl P-Hydroxycinnamate, cis-Pterostilbene, and (+)-Gallocatechin exhibited an increased number of cells in these active growth phases (Figure 5B). Importantly, the G0/G1 phase was significantly reduced in HGPS cultures treated with (+)-Pinoresinol, Bisdemethoxycurcumin, Pinosylvin, Methyl P-Hydroxycinnamate, and (+)-Gallocatechin, while the G2/M phase was substantially increased with (+)-Pinoresinol in HGPS cells and with Pinosylvin and (+)-Gallocatechin in control fibroblasts (Figure 5B). These treatments, therefore, effectively modified the cell cycle distribution in both the HGPS and control fibroblasts, significantly reducing the G0/G1 phase and increasing the S and G2/M phases, which indicates improved cellular proliferation and delayed senescence.

Supporting these observations, Western blot analyses, immunocytochemistry, and qPCR indicated reductions in the senescence markers, p16 and p21, following treatment with the selected compounds (Figure 5C–E, Figures S2 and S10). To compare the different models of control and HGPS cells, Western blot analysis was performed on mock-treated young control and HGPS cells (SNS < 5%) as well as old control and HGPS cells (SNS > 20%). The protein levels of p16 and p21 were determined and analyzed on the same membrane (Figure S7). As expected, the p16 and p21 levels increase with aging in both the control and HGPS cells, with HGPS fibroblasts showing significantly higher levels of these proteins in young cultures relative to their control counterparts (Figures S7A–C and S9M–O). In control fibroblasts, the p16 protein levels significantly decreased across all treatments, while the p21 levels were particularly reduced with treatments of Pinosylvin, Methyl P-Hydroxycinnamate, cis-Pterostilbene, and (+)-Gallocatechin (Figure 5C–E). Although changes in the p16 or p21 levels were not statistically significant in the HGPS groups, a trend toward decreased p21 levels was observed with the treatments of Pinosylvin, Methyl P-Hydroxycinnamate, cis-Pterostilbene, and (+)-Gallocatechin (Figure 5C–E). The mRNA levels of p16 and p21 generally tended to be lower across all treatments in the control and HGPS fibroblasts (Figure S10A,B). Importantly, HGPS fibroblasts treated with Methyl P-Hydroxycinnamate, cis-Pterostilbene, and (+)-Gallocatechin showed a significant reduction in the p16 levels (Figure S10A). Hence, the p21 levels were significantly lower in the HGPS cells treated with cis-Pterostilbene- and (+)-Gallocatechin (Figure S10B). Immunocytochemistry also revealed significant reductions in the p21-positive nuclei, with decreases ranging from 2.2% to 4.6% in both the control and HGPS fibroblasts under all treatment conditions (Figure S2).

Collectively, these findings demonstrate that botanical compounds, including Resveratrol, Bisdemethoxycurcumin, Pinosylvin, Methyl P-Hydroxycinnamate, cis-Pterostilbene, and (+)-Gallocatechin, significantly enhance growth rates and decrease cellular senescence in both control and HGPS fibroblasts. This effect is further supported by the increased progression through the S and G2/M phases of the cell cycle, suggesting that these compounds confer a protective effect and delay the onset of cellular aging in both normal and HGPS fibroblasts.

### 3.5. Modulation of the JAK-STAT Signaling by Resveratrol, Bisdemethoxycurcumin, cis-Pterostilbene, and (+)-Gallocatechin

**Figure 6:**
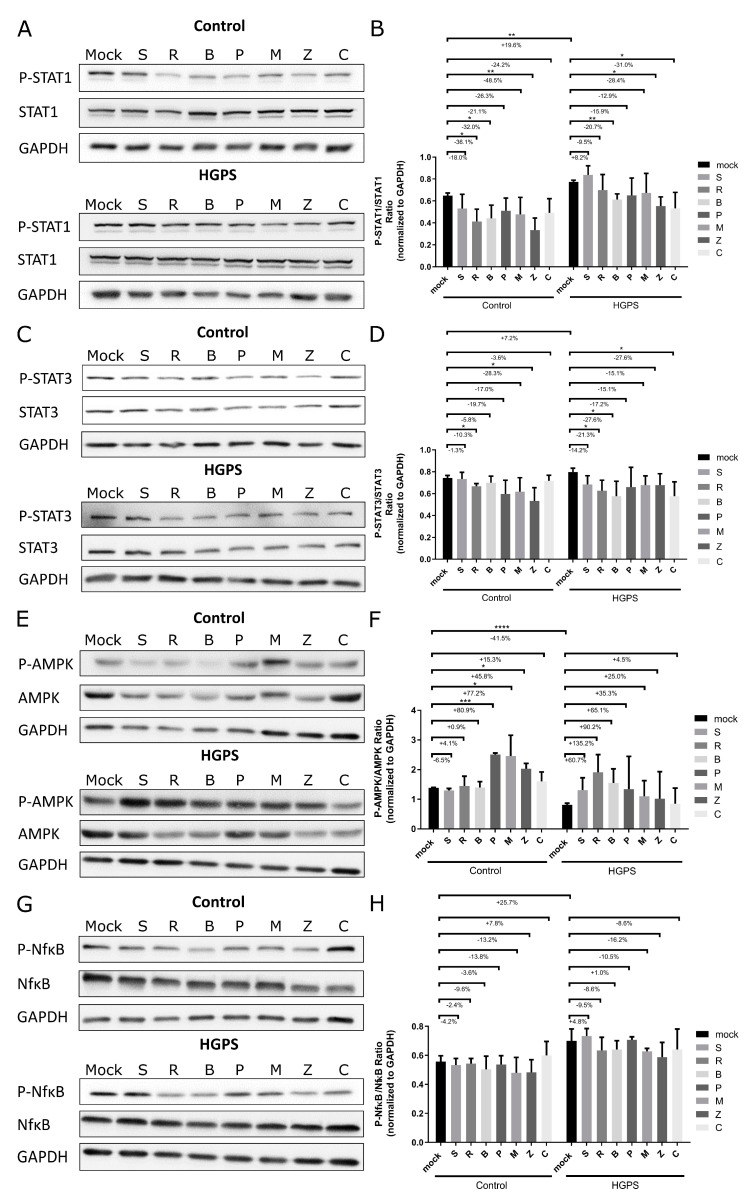
Western blot analysis of control and HGPS fibroblasts treated with botanical compounds. Control fibroblasts (5757C, 5567A, F369, M368) and HGPS fibroblasts (P003, P127, P271) with a senescence level of approximately 15% were treated for 7 days with no compound (mock), 2.5 μM (+)-Pinoresinol (S), 10 μM Resveratrol (R), 2 μM Bisdemethoxycurcumin (B), 1 μM Pinosylvin (P), 1.5 μM Methyl P-Hydroxycinnamate (M), 2.5 μM cis-Pterostilbene (Z), and 10 μM (+)-Gallocatechin (C). Panels (**A**,**C**,**E**,**G**) show representative Western blot images for phosphorylated and total forms of STAT1 (**A**), STAT3 (**C**), AMPK (**E**), and NFκB (**G**) from three experiments (n = 3). Panels (**B**,**D**,**F**,**H**) depict the ratios of phosphorylated to total STAT1 (**B**), STAT3 (**D**), AMPK (**F**), and NFκB (**H**) in both control and HGPS fibroblasts. Original western blots can be found at Figure S3. Graphs present mean ± SD; significance indicated by * *p* < 0.05; ** *p* < 0.01; *** *p* < 0.001; and **** *p* < 0.0001, using an unpaired *t*-test and one-way anova.

The JAK-STAT and NF-κB pathways play crucial roles in mediating inflammatory responses and are commonly upregulated during replicative senescence [20,46,47,48]. To investigate how selected botanical compounds might modulate these pathways and to assess their effects, we performed comprehensive analyses of key signaling molecules. This included quantifying the ratios of phosphorylated to total forms of STAT1 (P-STAT1/STAT1), STAT3 (P-STAT3/STAT3), and NFκB (P-NFκB/NFκB) in protein extracts from control and HGPS fibroblasts, which exhibited a baseline senescence level of approximately 15% prior to the initiation of treatments (Figure 6).

To examine the differences between control and HGPS fibroblasts, Western blot analysis comparing mock-treated groups from both control and HGPS fibroblasts was performed, and the ratios of P-STAT1/STAT1, P-STAT3/STAT3, P-AMPK/AMPK, and P-NfκB/NfκB in protein extracts were examined (Figure S8). As anticipated, the phosphorylated forms of STAT1, STAT3, and NfκB were increased in the HGPS fibroblasts, and the ratio of phosphorylated AMPK to total AMPK was reduced in mock-treated HGPS cells compared to mock-treated control fibroblasts (Figure S8A–H).

The fibroblasts were treated for 7 days with either no compound (mock) or with specific compounds including 2.5 μM (+)-Pinoresinol, 10 μM Resveratrol, 2 μM Bisdemethoxycurcumin, 1 μM Pinosylvin, 1.5 μM Methyl P-Hydroxycinnamate, 2.5 μM cis-Pterostilbene, and 10 μM (+)-Gallocatechin. The analyses indicated a general trend of decreased P-STAT1/STAT1 and P-STAT3/STAT3 ratios across all treatments, with significant reductions in control fibroblasts treated with Resveratrol (−36.1%), Bisdemethoxycurcumin (−32%), and cis-Pterostilbene (−48.5%). Similarly, HGPS cells showed significant decreases with Bisdemethoxycurcumin (−20.7%), cis-Pterostilbene (−28.4%), and (+)-Gallocatechin (−31%), with Resveratrol notably reducing the P-STAT3/STAT3 ratio in both control (−10.3%) and HGPS cells (−21.3%) (Figure 6A–D).

While phosphorylated NFκB levels were not significantly decreased, there was a reduction in the P-NFκB/NFκB ratios following treatments with (+)-Pinoresinol, Resveratrol, Bisdemethoxycurcumin, Pinosylvin, Methyl P-Hydroxycinnamate, and cis-Pterostilbene in both control and HGPS groups, indicating a tendency towards decreased P-NFκB levels, particularly in HGPS cells treated with Resveratrol, Bisdemethoxycurcumin, Methyl P-Hydroxycinnamate, cis-Pterostilbene, and (+)-Gallocatechin (Figure 6G,H). Quantitative real-time PCR analysis of NFκB mRNA expression levels confirmed these findings (Figure S10C). Although there were no statistically significant reductions in NFκB levels in either control or HGPS groups, treatments with (+)-Pinoresinol, Resveratrol, Bisdemethoxycurcumin, Pinosylvin, cis-Pterostilbene, and (+)-Gallocatechin tended to reduce NFκB expression in both control and HGPS fibroblasts (Figure S10C).

Given the pivotal role of AMPK signaling in regulating energy balance and its interactions with inflammatory pathways [49,50,51], we also examined its activation status. AMPK was significantly activated in control cells by treatments with Resveratrol, Bisdemethoxycurcumin, Pinosylvin, Methyl P-Hydroxycinnamate, cis-Pterostilbene, and (+)-Gallocatechin, particularly with Pinosylvin (+80.9%), Methyl P-Hydroxycinnamate (+77.2%), and cis-Pterostilbene (+45.8%), as shown by the elevated levels of P-AMPK (Figure 6E,F). This analysis highlights the broad impact of these botanical compounds on key cellular signaling pathways associated with inflammation and energy regulation.

Collectively, these results indicate that Resveratrol, Bisdemethoxycurcumin, Pinosylvin, Methyl P-Hydroxycinnamate, cis-Pterostilbene, and (+)-Gallocatechin effectively modulate key inflammatory and energy-regulating pathways, reducing the JAK-STAT and NF-κB activity while activating the AMPK signaling. This contributes to decreased inflammation and enhanced energy regulation in both control and HGPS fibroblast treatments.

### 3.6. Reduced Reactive Oxygen Species (ROS) and Enhanced Autophagy Activity upon Treatment with Botanical Compounds

**Figure 7:**
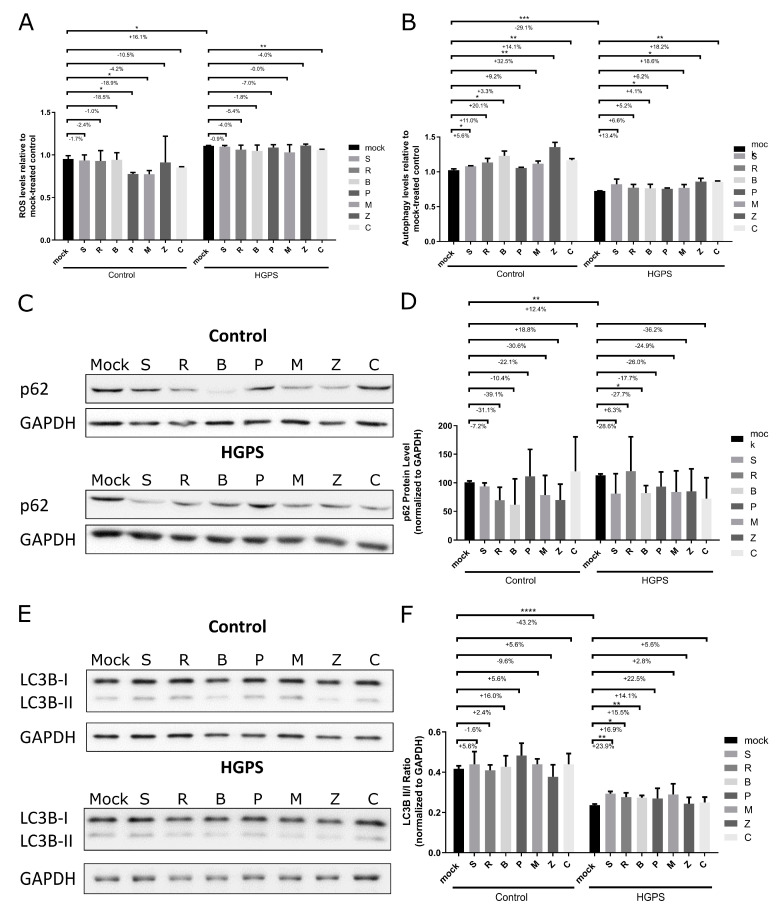
Assessment of ROS and autophagy levels in control and HGPS Fibroblasts. Control fibroblasts (5757C, 5567A, F369, M368) and HGPS fibroblasts (P003, P127, P271) with a senescence level of approximately 15% were treated for 7 days with no compound (mock) and with varying concentrations of the following botanical compounds: 2.5 μM (+)-Pinoresinol (S), 10 μM Resveratrol (R), 2 μM Bisdemethoxycurcumin (B), 1 μM Pinosylvin (P), 1.5 μM Methyl P-Hydroxycinnamate (M), 2.5 μM cis-Pterostilbene (Z), and 10 μM (+)-Gallocatechin (C). (**A**) Intracellular ROS levels were determined by measuring oxidized dichlorofluorescein (DCF) using the DCFDA cellular ROS detection assay (n = 3). (**B**) Autophagy levels were analyzed by measuring monodansylcadaverine (MDC) via fluorescence photometry (n = 3). (**C**,**E**) Representative Western blot images for p62 (**C**) and LC3B (**E**). (**D**) Quantification of p62 protein levels normalized to GAPDH (n = 3). Original western blots can be found at Figure S4. (**F**) Ratio of LC3B-II to LC3B-I (n = 3). Graphs show mean ± SD; significance levels are indicated by * (*p* < 0.05), ** (*p* < 0.01), *** (*p* < 0.001), and **** (*p* < 0.0001), analyzed using an unpaired *t*-test and one-way anova.

Previous studies have shown that mitochondrial dysfunction and altered reactive oxygen species (ROS) levels are key contributors to the cellular and metabolic defects, including changes in autophagy, senescence, and DNA damage in HGPS and other aging-related contexts [49,50,51]. In this study, we assessed the cellular oxidative stress using the DCFDA cellular ROS detection assay (Figure 7A). Consistent with previous reports, ROS levels were higher in mock-treated HGPS cells compared to normal cells [52] (Figure 7A). We observed significant reductions in the ROS levels in control fibroblasts treated with Pinosylvin (−18.5%) and Methyl P-Hydroxycinnamate (−18.9%). In HGPS fibroblasts, treatment with (+)-Gallocatechin led to a reduction of 4.0% (Figure 7A).

DNA damage has profound cellular and metabolic implications, promoting premature senescence, cellular dysfunction, and inflammation and consequently, accelerating the aging process [53,54,55]. To investigate the impact of selected botanical compounds on DNA damage, we performed immunocytochemistry to detect y-H2AX, a biomarker for double-strand breaks [56] (Figure S6). Both control and HGPS fibroblasts showed significantly fewer y-H2AX positive nuclei following treatment with (+)-Pinoresinol, Resveratrol, Bisdemethoxycurcumin, Pinosylvin, Methyl P-Hydroxycinnamate, cis-Pterostilbene, and (+)-Gallocatechin. The reduction in DNA damage was more pronounced in control groups, with a 1.6–3.3% decrease in y-H2AX positive nuclei, while HGPS fibroblasts showed a reduction of 1.4–2.4% (Figure S6B).

Following the observed activation of AMPK signaling by certain botanical compounds, we assessed autophagy levels in control and HGPS fibroblasts, given AMPK’s role in autophagy regulation [57,58]. Autophagy was quantified using fluorescence photometry to measure monodansylcadaverine (MDC) levels, supported by Western blot analyses of p62 (SQSTM1) and LC3B, established markers of autophagy [59,60,61] (Figure 7B–F). Enhanced autophagy was observed in both control and HGPS fibroblasts treated with botanical compounds, with a significant increase in controls treated with (+)-Pinoresinol (+4.1%), Bisdemethoxycurcumin (+20.1%), cis-Pterostilbene (+32.5%), and (+)-Gallocatechin (+14.1%). Similarly, HGPS cells treated with Pinosylvin (+4.1%), cis-Pterostilbene (+18.6%), and (+)-Gallocatechin (+18.2%) showed a significant autophagy increase (Figure 7B).

In addition, we quantified the levels of p62 and the LC3B II/I ratio to assess autophagy activity more precisely, as a reduction in p62 indicates effective autophagic cargo clearance, and an increase in the LC3B II/I ratio suggests enhanced autophagosome formation [62,63]. To investigate the differences between control and HGPS fibroblasts, we first performed a Western blot analysis on young control (SNS < 5%), old control (SNS > 20%), young HGPS (SNS < 5%), and old HGPS (SNS > 20%) fibroblasts. All samples were loaded onto the same membrane for direct comparison, and the protein levels of LC3B I and II and p62 were analyzed (Figures S7 and S9A–C). As expected, the LC3B II to I ratio decreased with age (Figure S7D,E). Old HGPS fibroblasts exhibited significantly lower ratios of LC3B II to I compared to old control cells (Figure S7E). This was further supported by the mock-treated groups, where HGPS fibroblasts without treatment showed a reduced LC3B II/I ratio and increased p62 protein levels (Figure S9A–C).

Western blot analyses showed a significant (27.7%) reduction in the p62 levels in Bisdemethoxycurcumin-treated HGPS cells (Figure 7C,D), with other compounds also showing a trend towards decreased p62 levels, though these were not statistically significant (Figure 7C,D). As previously reported, the LC3B II/I ratio was significantly lower in HGPS cells compared to normal cells (Figure 7E,F). Remarkably, this ratio increased in all treated HGPS groups, particularly following (+)-Pinoresinol (+23.9%), Resveratrol (+16.9%), and Bisdemethoxycurcumin (+15.5%) treatments (Figure 7E,F). In contrast, control cells showed only moderate increases in the LC3B II/I ratios with treatments such as (+)-Pinoresinol, Bisdemethoxycurcumin, Pinosylvin, Methyl P-Hydroxycinnamate, and (+)-Gallocatechin (Figure 7E,F).

Overall, these results indicate that botanical compounds not only enhance autophagy in normal (control) fibroblasts but also exert more pronounced effects in HGPS cells, where autophagy is especially impaired [53]. In control cells, treatments such as (+)-Pinoresinol, Bisdemethoxycurcumin, cis-Pterostilbene, and (+)-Gallocatechin led to moderate increases in LC3B II/I ratios, indicating improved autophagic activity. This enhancement was even more significant in HGPS fibroblasts, evidenced by substantial increases in LC3B II/I ratios and decreases in p62 levels, particularly with treatments like (+)-Pinoresinol, Resveratrol, and Bisdemethoxycurcumin.

### 3.7. Summary of Botanical Compound Screening for Impact on Cellular Functions in Normal and HGPS Fibroblasts

**Figure 8:**
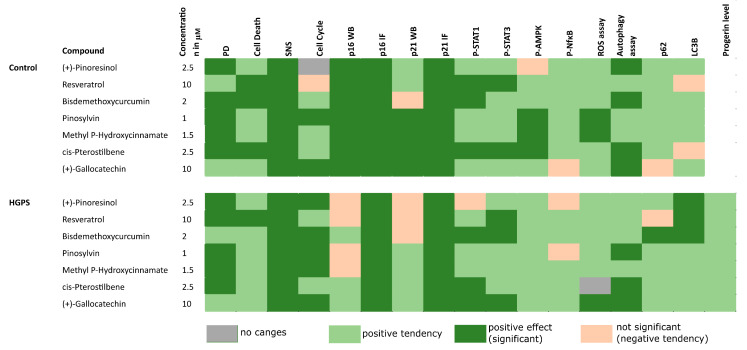
Heatmap illustrates the effects of botanical compounds on various cellular functions and pathways in control and HGPS fibroblasts. Dark green indicates significant amelioration, light green represents non-significant amelioration, orange denotes a non-significant negative effect, and grey indicates no change.

To summarize, this study highlights the efficacy of specific botanical compounds in modulating cellular functions with a focus on their impact on normal and premature aging fibroblasts (Figure 8). Key findings reveal that compounds, such as Resveratrol, Bisdemethoxycurcumin, and cis-Pterostilbene, significantly lower the levels of P-STAT1 and P-STAT3, markers of the JAK1 and JAK2 signaling pathways that are closely linked to cellular aging and inflammation processes. These compounds effectively reduce signs of cellular aging and enhance cell proliferation during long-term treatments.

In control fibroblasts, treatments with Resveratrol, Bisdemethoxycurcumin, and cis-Pterostilbene resulted in significant reductions in senescence markers and increases in cell proliferation and autophagy. The effects were even more pronounced in HGPS cells, where these compounds not only significantly reduced P-STAT1 and P-STAT3 levels but also substantially enhanced autophagy, as indicated by increased LC3B II/I ratios and decreased p62 levels. The enhancement of autophagy was particularly significant in HGPS cells treated with Resveratrol and Bisdemethoxycurcumin, highlighting their potential therapeutic benefits in conditions characterized by accelerated aging.

## 4. Discussion

Aging continues to be a central focus of contemporary health science, with various strategies being developed to extend life expectancy and improve the quality of life, particularly in areas such as skin health and age-related alterations [8,54,55]. Botanical compounds and plant extracts are increasingly recognized for their beneficial properties, including anti-inflammatory and antioxidant effects. These properties make them promising candidates for skin care and anti-aging treatments [56,64,65].

In this study, botanical compounds were screened in silico for their potential to mimic the effects of baricitinib, a promising JAK/STAT inhibitor known to ameliorate cellular senescence and improve overall cellular homeostasis [19,20]. Several botanical compounds were screened and evaluated for their effects on JAK-STAT inhibition, cell proliferation, and cytotoxicity. Notably, most treatments administered at high doses or over an extended period of 7 days resulted in cytotoxic effects and diminished cell proliferation. These observations align with prior research indicating a dose-dependent cytotoxicity with resveratrol in cancer cells, where even concentrations as low as 50 µM significantly reduced cell proliferation [66,67,68]. Additionally, Giovannelli et al. found that while a 7-day treatment with 50 µM resveratrol inhibited fibroblast proliferation, a lower concentration of 5 µM did not produce adverse effects [69]. Similar trends of growth inhibition and cytotoxicity at high doses over prolonged periods have also been reported for Bisdemethoxycurcumin, (+)-Taxifolin, and Epigallocatechin [70,71,72,73,74]. In the context of cancer therapy, high-dose Pterostilbene treatments are known to suppress cancer cell proliferation [75,76]. Specifically, diffuse large B-cell lymphoma cells treated with Pterostilbene at concentrations ≥50 µM showed reduced growth and viability, with cell death induced through the inhibition of ERK1/2 and the activation of the p38MAPK signaling pathways [77].

Finally, seven botanical compounds were identified that effectively inhibit the JAK/STAT pathway without inducing cytotoxic effects at low doses: (+)-Pinoresinol, Resveratrol, Bisdemethoxycurcumin, Pinosylvin, Methyl P-Hydroxycinnamate, cis-Pterostilbene, and (+)-Gallocatechin. Subsequent analyses revealed that these compounds, particularly Resveratrol, Bisdemethoxycurcumin, Pinosylvin, Methyl P-Hydroxycinnamate, cis-Pterostilbene and (+)-Gallocatechin, were especially beneficial in improving age-related effects in both normal and HGPS fibroblasts. Notably, certain compounds demonstrated broad impacts across various cellular pathways. Cis-Pterostilbene, Bisdemethoxycurcumin, Pinosylvin, Methyl P-Hydroxycinnamate, and (+)-Gallocatechin modulated cell proliferation, senescence, the JAK/STAT pathway, the AMPK and NfκB pathway, and autophagy in aging dermal fibroblasts.

Given the complex nature of aging—which encompasses genomic defects, telomere attrition, epigenetic changes, autophagy disruption, cellular senescence, mitochondria dysfunction, and chronic inflammation—comprehensive and extensive treatments are needed [55]. Among the tested compounds, Pterostilbene stood out in normal fibroblasts, significantly reducing senescence, inhibiting the JAK/STAT pathway, and enhancing both proliferation and AMPK signaling. In HGPS fibroblasts, Bisdemethoxycurcumin and (+)-Gallocatechin were the most effective in inhibiting the JAK/STAT pathway and also significantly improved cell proliferation, senescence, ROS levels, and autophagy. Overall, seven botanical compounds were found to suppress the JAK/STAT signaling pathway and substantially reduce age-related cellular changes in aging fibroblasts without inducing cytotoxic effects at low doses.

Chronic inflammation, an important hallmark of aging, negatively affects the skin by damaging cells, altering cellular functions, and inducing cellular senescence [55,78,79]. Pterostilbene and Bisdemethoxycurcumin are well known as anti-inflammatory agents that reduce pro-inflammatory cytokines like interleukin-6 (IL-6) and TNF-α [80,81]. This low-grade, chronic inflammation, often referred to as “inflammaging”, is prevalent during aging, with many inflammatory mediators and markers of senescence upregulated in old individuals [13,82]. Treatments with Pterostilbene and Bisdemethoxycurcumin effectively inhibited the JAK/STAT pathway, which is closely associated with inflammation [20,83], and reduced senescence levels. Similarly, Resveratrol, Pinosylvin, Methyl P-Hydroxycinnamate, and (+)-Gallocatechin also demonstrated reductions in P-STAT1, P-STAT3, and senescence levels in both normal and HGPS fibroblasts.

As senescent cells accumulate, they contribute to the aging process by reducing cell proliferation and increasing inflammation, highlighting that targeting senescence is a promising anti-aging strategy [84,85]. Senescent cells are characterized by cell cycle arrest and produce a senescence-associated secretory phenotype (SASP), which is beneficial for tissue regeneration and inflammation management in healthy individuals but contributes to chronic inflammation and altered stem cell renewal during aging [84,86,87,88,89]. Besides directly targeting the senescence cells, the activation of AMPK was shown to delay cellular senescence [90]. AMPK is involved in regulating cellular energy metabolism, autophagy, and senescence [91,92]. Activation of AMPK has demonstrated a reduction in cellular senescence and the levels of SASP-related pro-inflammatory mediators in primary human skin fibroblasts, with supporting evidence from rodent studies [88,93,94].

In this study, we showed that treatments with Pinosylvin, Methyl P-Hydroxycinnamate, and cis-Pterostilbene effectively activated AMPK in control fibroblasts, reducing senescence. AMPK activation is closely linked to autophagy, which plays an important role in maintaining cellular homeostasis. Dysregulation in autophagy is associated with aging phenotypes and age-related diseases [31,95,96,97,98,99]. Autophagy also plays an important role during dermal aging, influencing key cellular changes and leading to alterations in keratinocytes, skin fibroblasts, and melanocytes [100]. The decline in autophagy activation with age impacts skin health; however, restoring this function has been shown to extend health and lifespan [99,100]. The importance of autophagy in skin physiology is further shown by a mouse model deficient in the autophagy-related gene 7 (Atg7), which underscored the essential role of autophagy in keratinocyte proliferation and differentiation [101]. Moreover, the induction of autophagy has been proven effective in ameliorating skin aging and enhancing keratinocyte differentiation, establishing it as a potential therapeutic target in dermatological aging [102,103].

In control fibroblasts, treatments with Bisdemethoxycurcumin, cis-Pterostilbene, and (+)-Gallocatechin significantly improved autophagy. Similar ameliorations were observed with Resveratrol and Pinosylvin, further validating the beneficial effects of these botanical compounds in promoting autophagy and potentially counteracting aging-related changes in skin cells. Pterostilbene and Resveratrol are particularly effective in enhancing autophagy, with Pterostilbene having greater bioavailability than Resveratrol. Additionally, previous studies have noted the autophagy-enhancing effects of Bisdemethoxycurcumin [104,105,106,107,108]. Taken together, these findings suggest that treatment with the botanical compounds Bisdemethoxycurcumin, Resveratrol, Pinosylvin, cis-Pterostilbene, and (+)-Gallocatechin effectively activates autophagy. Therefore, these compounds represent promising strategies for anti-aging therapy, especially skin anti-aging.

## 5. Conclusions

In this investigation, we demonstrated the beneficial effects of the botanical compounds (+)-Pinoresinol, Resveratrol, Bisdemethoxycurcumin, Pinosylvin, Methyl P-Hydroxycinnamate, cis-Pterostilbene, and (+)-Gallocatechin on aging fibroblasts. Particularly, Bisdemethoxycurcumin, Pinosylvin, Methyl P-Hydroxycinnamate, cis-Pterostilbene, and (+)-Gallocatechin have shown broad effectiveness, impacting various cell functions and pathways. Although further studies, including in vivo experiments, are necessary to validate these findings, the use of these botanical compounds as supplements or treatments could offer significant anti-aging benefits and help mitigate age-related changes.

## Figures and Tables

**Table 1:** Passage numbers and senescence levels for each fibroblast primary cell strain.

Cell Strain	Passage Number	Senescence
GM05757C	Passage 19–23	11.3–14.5%
GM05567A	Passage 20–24	11.5–13.2%
HGFDFN369	Passage 19–21	11.6–13.3%
HGMDFN368	Passage 19–21	11.1–14.2%
GM01651C	Passage 19–23	12.6–13.5%
HGADFN003	Passage 16–21	12.2–14.9%
HGADFN164	Passage 19–21	12.2–23.3%
HGADFN271	Passage 15–16	10.1–13.4%
HGADFN127	Passage 16–17	12.1–12.9%

**Table 2:** Top phytochemicals that bound to the ATP-binding site of JAK1/2 with a docking score above −6.0 kcal/mol and exhibited high drug-likeliness.

NPC ID	Phytochemical Name	Structure	Docking Score for JAK-2_XP	Docking Score for JAK-1_XP	Pubchem ID	Linpinski Rule of 5 Violation *	Molecular Weight	Log P	Hbond Acceptor	Hbond Donor
NPC40258	(3S,5S)-1,7-Bis(4-Hydroxyphenyl)Heptane-3,5-Diol [Hannokinol]	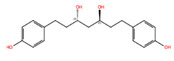	−10.08	−10.02	10335921	0	316.40	2.78	4.90	4
NPC177576 **	Platyphyllone	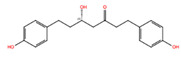	−9.42	−9.57	13347313	0	314.38	3.43	4.20	2
NPC38041	(−)-Pinoresinol 4-O-Beta-D-Glucopyranoside	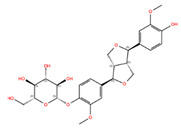	−9.38	−6.03	11168362	2	520.53	0.82	14.9	5
NPC42760 **	(2S,3S)-2-(3,4,5-Trihydroxy-Phenyl)-Chroman-3,5,7-Triol	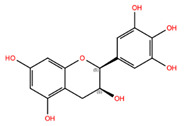	−9.17	−9.88	10425234	1	306.27	−0.19	6.20	6
NPC70843 **	1,2-Dihydrobis(De-O-Methyl)-Curcumin	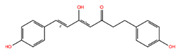	−8.90	−4.92	10614892	0	310.35	3.45	3.25	2
NPC78119 **	(3S)-1,7-Bis(4-Hydroxyphenyl)-(6E)-6-Hepten-3-Ol	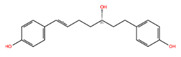	−8.87	−7.12	38362126	0	298.38	3.68	3.20	3
NPC68269	Bisdemethoxycurcumin	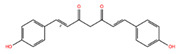	−8.42	−8.39	5315472	0	308.33	2.41	5,50	2
NPC130193 **	(1E,4Z,6E)-5-Hydroxy-1,7-Bis(4-Hydroxyphenyl)Hepta-1,4,6-Trien-3-One	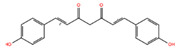	−8.32	−7.65	5324473	0	308.33	2.54	5.50	2
NPC23402 **	(3S)-Methoxy-1,7-Bis(4-Hydroxyphenyl)-6E-Hepten-5-One	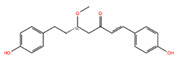	−8.32	−8.03	11723901	0	326.39	3.46	5.20	2
NPC15658	(−)-Catechin	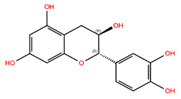	−8.08	−8.40	73160	0	290.27	0.44	5.45	5
NPC246162	(+)-Taxifolin	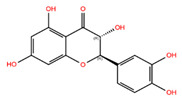	−8.04	−11.09	439533	0	304.26	0.08	6.45	4
NPC220825	(−)-Epigallocatechin	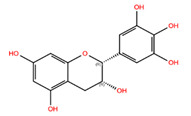	−7.76	−10.79	72277	1	306.27	−0.19	6.20	6
NPC219876	Cianidanol	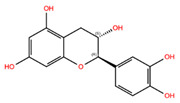	−7.64	−9.05	9064	0	290.27	0.46	5.45	5
NPC268342	(+)-Gallocatechin	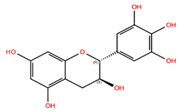	−7.51	−9.31	65084	1	306.27	−0.20	6.20	6
NPC161571	Resveratrol	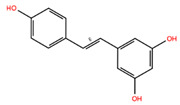	−7.49	−8.52	445154	0	228.25	1.99	2.25	3
NPC228346	4,4′-((1S,3Ar,4S,6Ar)-Hexahydrofuro [3,4-C]Furan-1,4-Diyl)Bis(2-Methoxyphenol) [(+)-Pinoresinol]	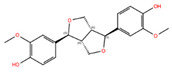	−7.20	−10.07	73399	0	358.39	2.89	6.40	2
NPC69332	1,7-Bis(4-Hydroxyphenyl)Hepta-4E,6E-Dien-3-One	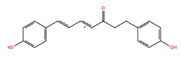	−7.12	−7.80	10613719	0	294.35	3.56	3.50	2
NPC291789	Pinosylvin	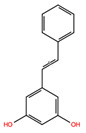	−6.55	−7.35	5280457	0	212.25	3.10	1.50	2
NPC253746	Methyl P-Hydroxycinnamate	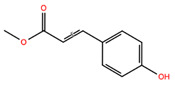	−6.39	−7.26	5319562	0	178.19	2.16	2.75	1
NPC228287	(Z)-4-(3,5-Dimethoxystyryl)Phenol [Pterostilbene]	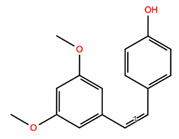	−6.39	−7.95	5320791	0	256.30	3.71	2.25	1
NPC17525 **	4,4′-Dihydroxychalcone	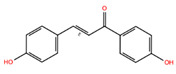	−6.29	−8.94	5467477	0	240.26	2.13	3.50	2

* Lipinski rule of 5 is a set of guidelines predicting the oral bioavailability of a compound. Per the rule, compounds are more likely to be orally active if they have a molecular weight <= 500 daltons, LogP <= 5, hydrogen bond acceptor <= 10, hydrogen bond donor <= 5. ** Compounds excluded from the study.

**Table 3:** Selected concentrations of the candidate botanical compounds.

Compound	Short Name	Concentration in μM
(+)-Pinoresinol	S	2.5
Resveratrol	R	10
Bisdemethoxycurcumin	B	2
Pinosylvin	P	1
Methyl P-Hydroxycinnamate	M	1.5
cis-Pterostilbene	Z	2.5
(+)-Gallocatechin	C	10

## Data Availability

Data are contained within the article and Supplementary Materials.

## Supplementary Materials

The following supporting information can be downloaded at https://www.mdpi.com/article/10.3390/biom14101310/s1: Figure S1: Full-length scan of Western blots from Figure 2; Figure S2: Immunocytochemistry for senescence marker p21 and vimentin in both control and HGPS fibroblasts; Figure S3: Full-length scan of Western blots from Figure 5 and Figure 6; Figure S4: Full-length scan of Western blots for Figure 7; Figure S5: Western blot analysis of HGPS fibroblasts treated with botanical compounds; Figure S6: Immunocytochemistry for DNA damage marker H2AX and Lamin B1 in both Control and HGPS fibroblasts; Figure S7: Western Blot Analysis of young and old Control and HGPS Fibroblasts without treatment; Figure S8: Western Blot Analysis of mock treated Control and HGPS Fibroblasts; Figure S9: Western Blot Analysis of mock treated Control and HGPS Fibroblasts; Figure S10: Replicative senescence levels profiles and NFκB expression of Control and HGPS fibroblasts under different compound treatment conditions; Table S1: Primers used for real-time quantitative PCR analysis.
