# Supplementary material for: Enhancing Cellular Homeostasis: Targeted Botanical Compounds Boost Cellular Health Functions in Normal and Premature Aging Fibroblasts
Source: Biomolecules. 2024 Oct 16;14(10):1310. doi: 10.3390/biom14101310 (PMC11506649; doi:10.3390/biom14101310)
Supplement: Supplementary file 1 [file biomolecules-14-01310-s001.zip › biomolecules-3190250-supplementary.pdf]

Article

Enhancing Cellular Homeostasis: Targeted Botanical Compounds Boost Cellular Health Functions in Normal and Premature Aging Fibroblasts

Ramona Hartinger [1], Khushboo Singh [2], Jesse Leverett [2] and Karima Djabali [1,*]

Supplementary Figures

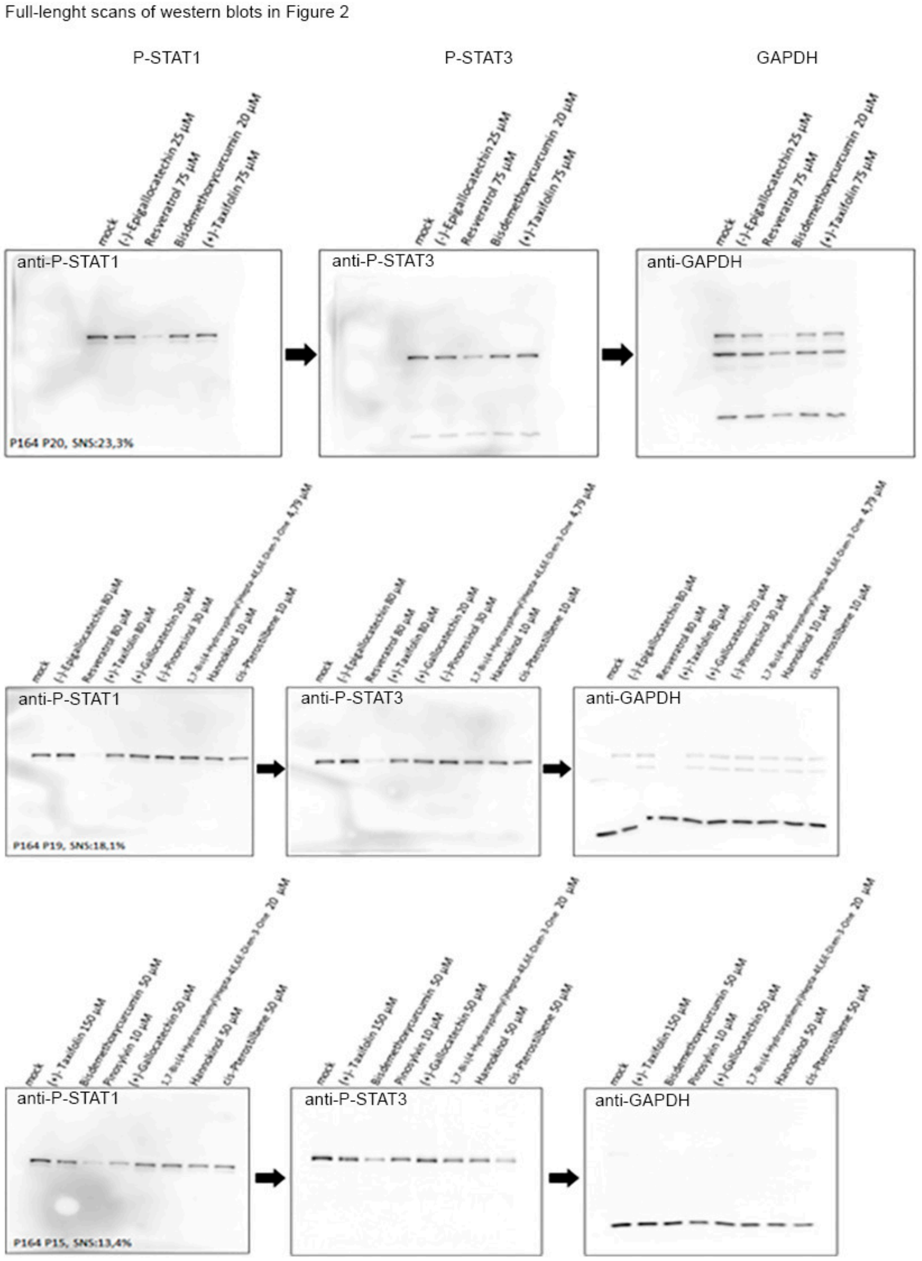

Figure S1. Full-length scan of western blots from Figure 2. Western blots of HGPS cells (HGADFN164) probed with anti-P-STAT1 (left scan), anti-P-STAT3 (middle scan) and anti-GAPDH (right scan).

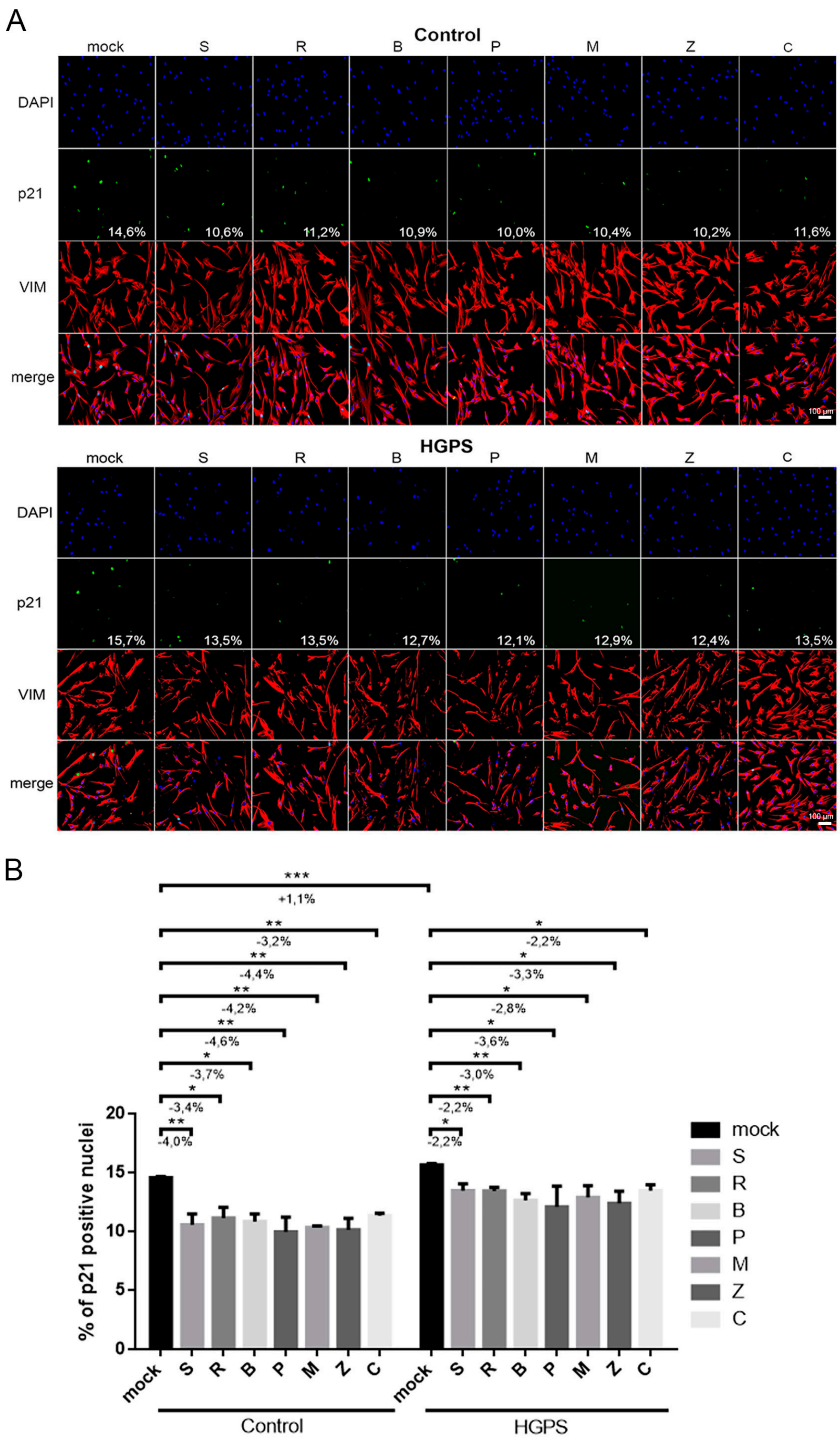

Figure S2: Immunocytochemistry for senescence marker p21 and vimentin in both Control and HGPS fibroblasts. Control fibroblasts (5757C, 5567A, F369, M368) and HGPS fibroblasts (P003, P127, P271) with a senescence level of ~15% were used and treated with no compound (mock), 2.5 µM (+)-Pinoresinol (S), 10 µM Resveratrol (R), 2 µM Bisdemethoxycurcumin (B), 1 µM Pinosylvin (P), 1.5 µM Methyl P-Hydroxycinnamate (M), 2.5 µM cis-Pterostilbene (Z) and 10 µM (+)-Gallocatechin (C) for 7d. (A) Representative images for immunocytochemistry for p21 and vimentin. Cells were counterstained with DAPI. Scale bar 100 µm. (B) Quantification of the number of p21 positive nuclei in Control and HGPS fibroblasts (n=3). Graphs show mean ± SD (n = 3); * $p < 0.05$; ** $p < 0.01$; *** $p < 0{,}001$; **** $p < 0.0001$; unpaired t-test and one-way anova.

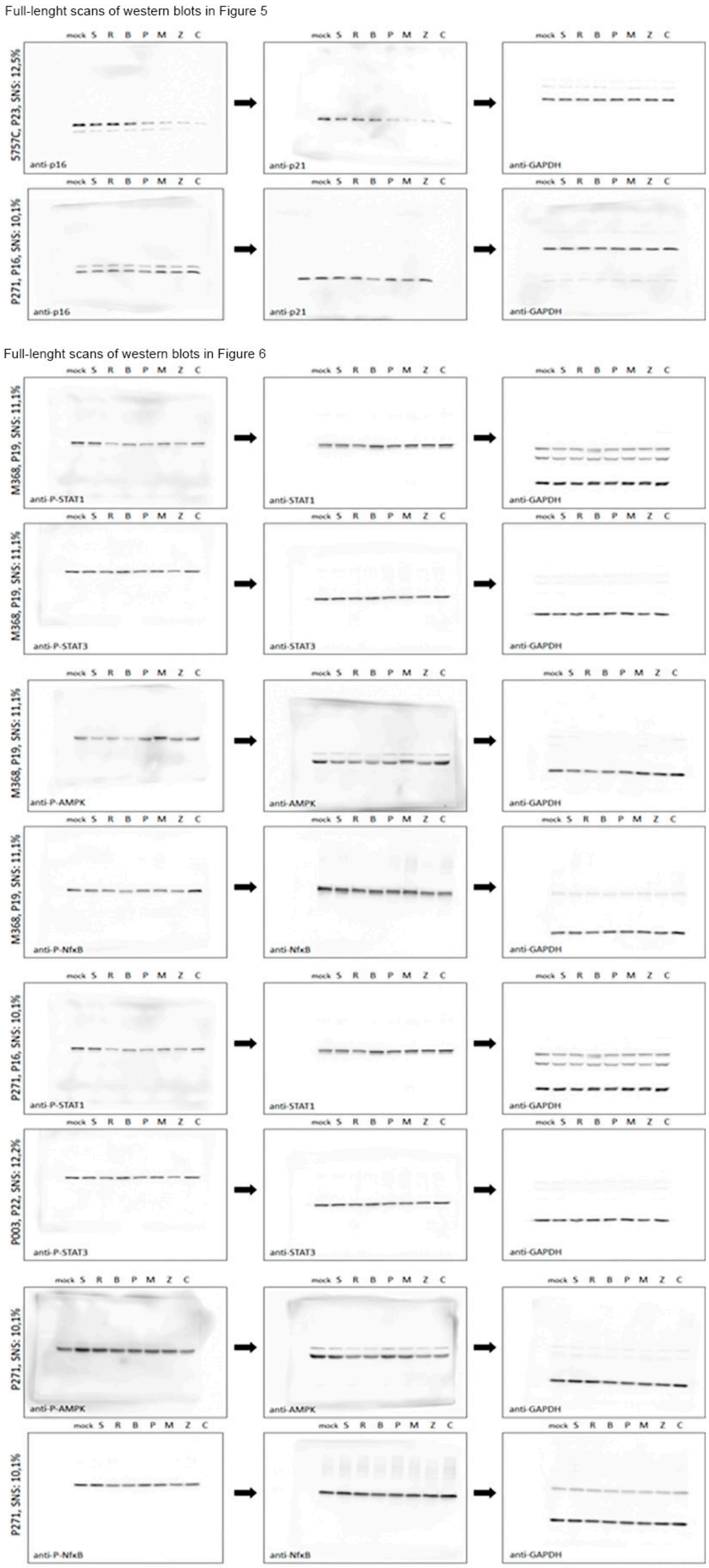

Figure S3: Full-length scan of western blots of figure 5 and figure 6

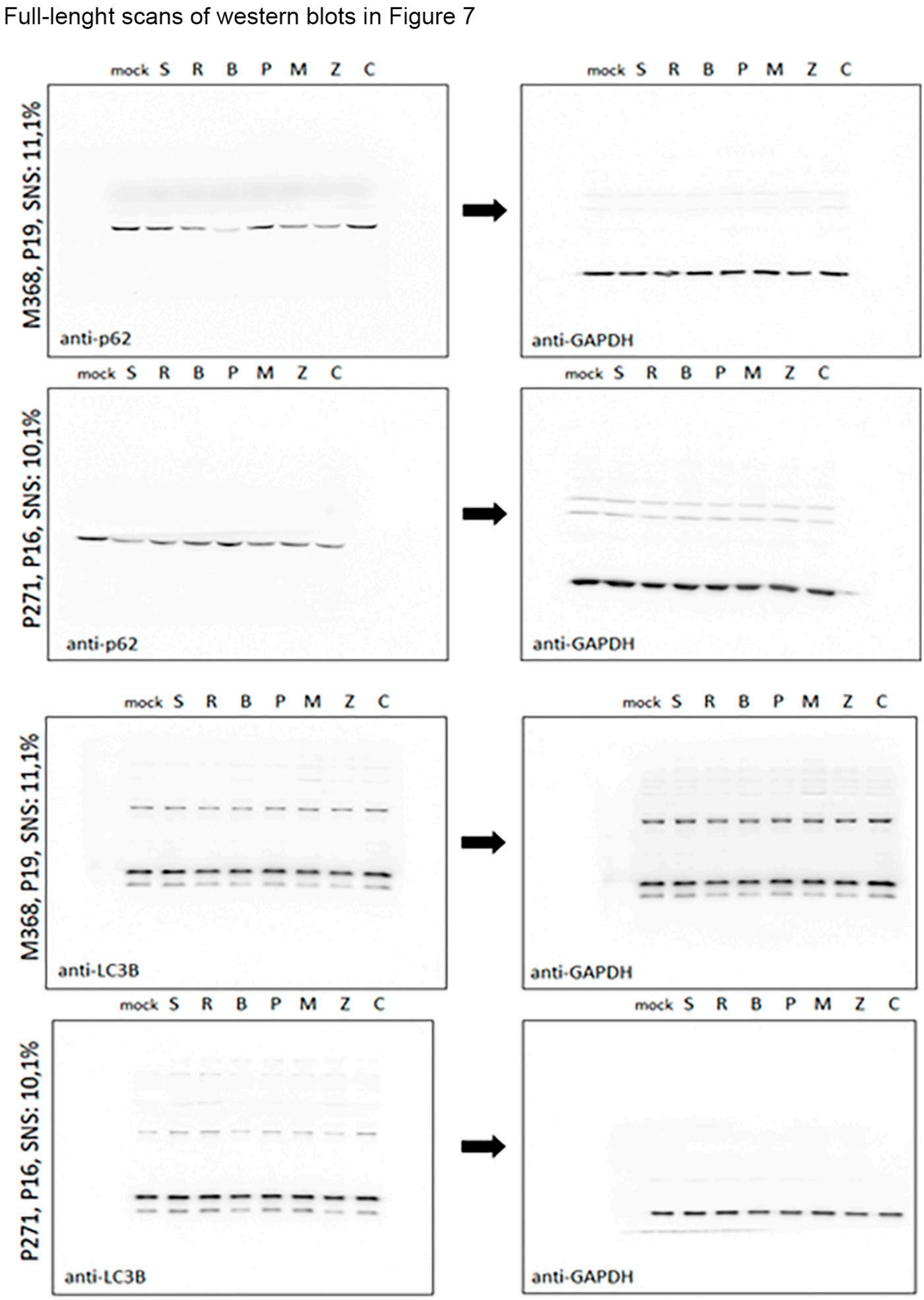

Figure S4: Full-length scan of western blots of figure 7

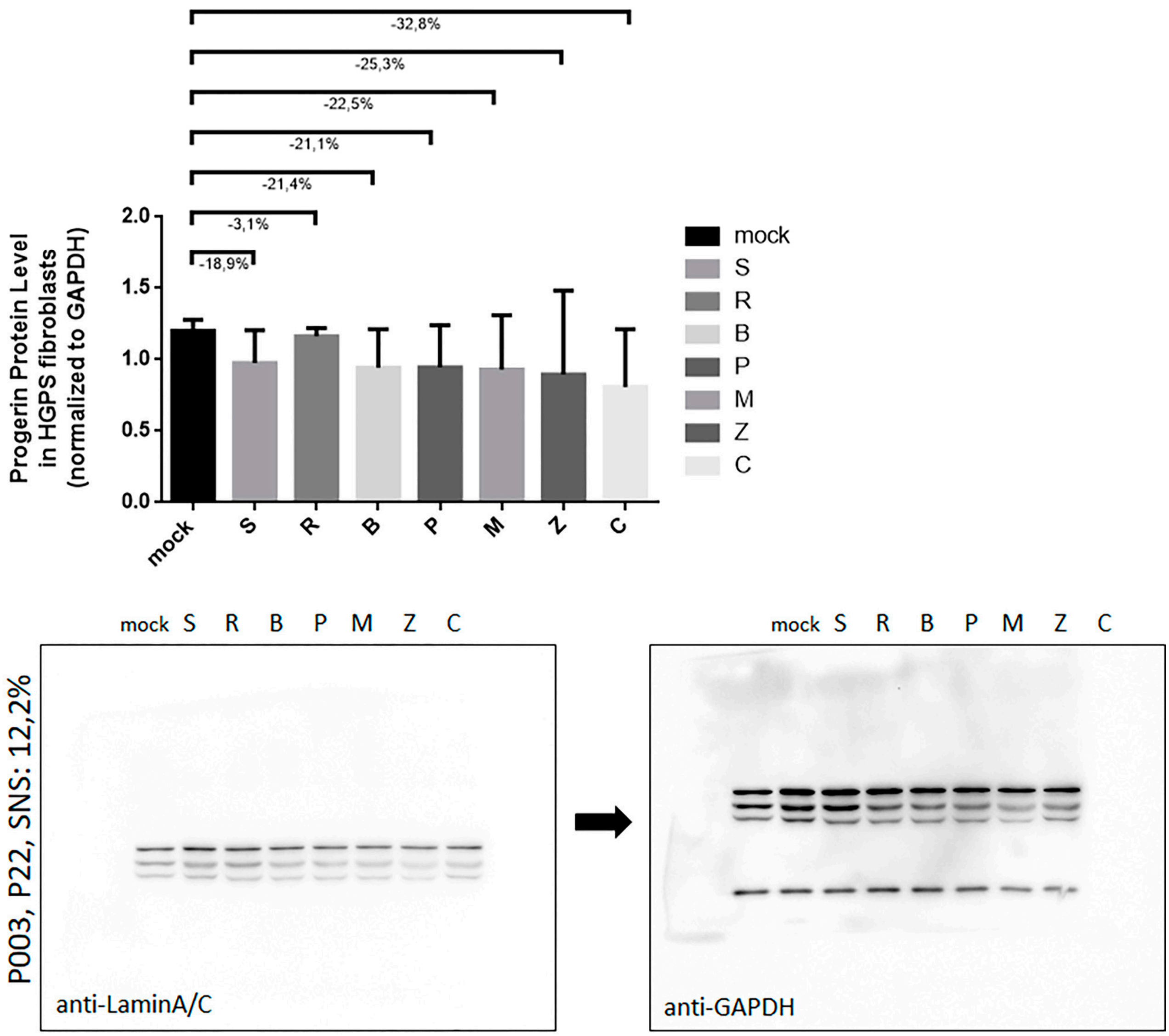

Figure S5: Western Blot Analysis of HGPS Fibroblasts treated with Botanical Compounds**.** HGPS fibroblasts (P003, P127, P271) with a senescence level of approximately 15% were treated for 7 days with no compound (mock), 2.5 µM (+)-Pinoresinol (S), 10 µM Resveratrol (R), 2 µM Bisdemethoxycurcumin (B), 1 µM Pinosylvin (P), 1.5 µM Methyl P-Hydroxycinnamate (M), 2.5 µM cis-Pterostilbene (Z), and 10 µM (+)-Gallocatechin (C). Panel A depict the Progerin protein level in HGPS fibroblasts. Graphs present mean ± SD; significance indicated by * $p < 0.05$; ** $p < 0.01$; *** $p < 0.001$; **** $p < 0.0001$, using an unpaired t-test and one-way anova. Panel B shows full-length scan of western blot representative for panel A.

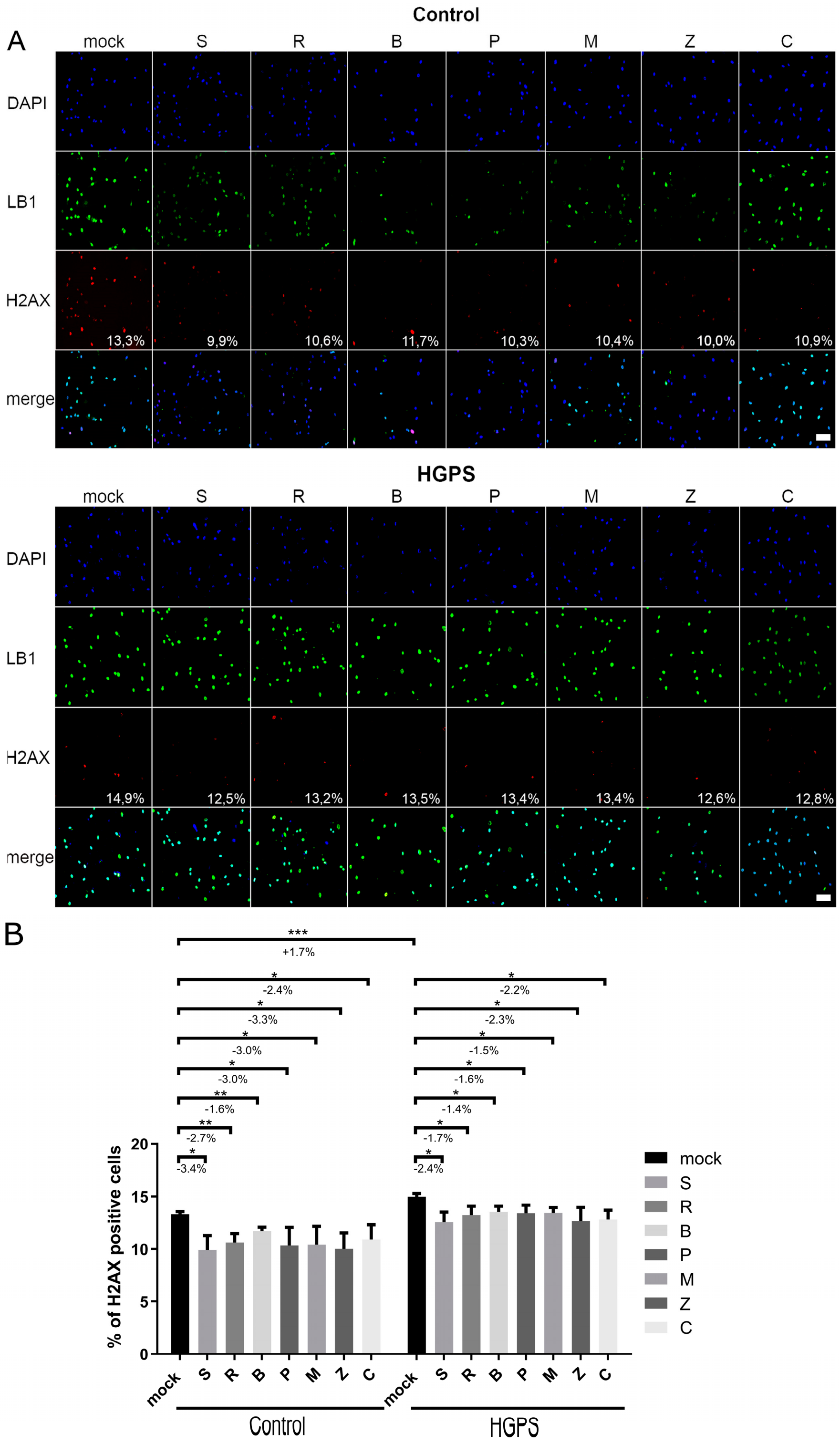

Figure S6: Immunocytochemistry for DNA damage marker H2AX and Lamin B1 in both Control and HGPS fibroblasts. Control fibroblasts (5757C, 5567A, F369, M368) and HGPS fibroblasts (P003, P127, P271) with a senescence level of ~15% were used and treated with no compound (mock), 2.5 µM (+)-Pinoresinol (S), 10 µM Resveratrol (R), 2 µM Bisdemethoxycurcumin (B), 1 µM Pinosylvin (P), 1.5 µM Methyl P-Hydroxycinnamate (M), 2.5 µM cis-Pterostilbene (Z) and 10 µM (+)-Gallocatechin (C) for 7d. (A) Representative images for immunocytochemistry for H2AX and Lamin B1. Cells were counterstained with DAPI. Scale bar 100 µm. (B) Quantification of the number of H2AX positive nuclei in Control and HGPS fibroblasts (n=3). Graphs show mean ± SD (n = 3); * $p < 0.05$; ** $p < 0.01$; *** $p < 0,001$; **** $p < 0.0001$; unpaired t-test and one-way anova.

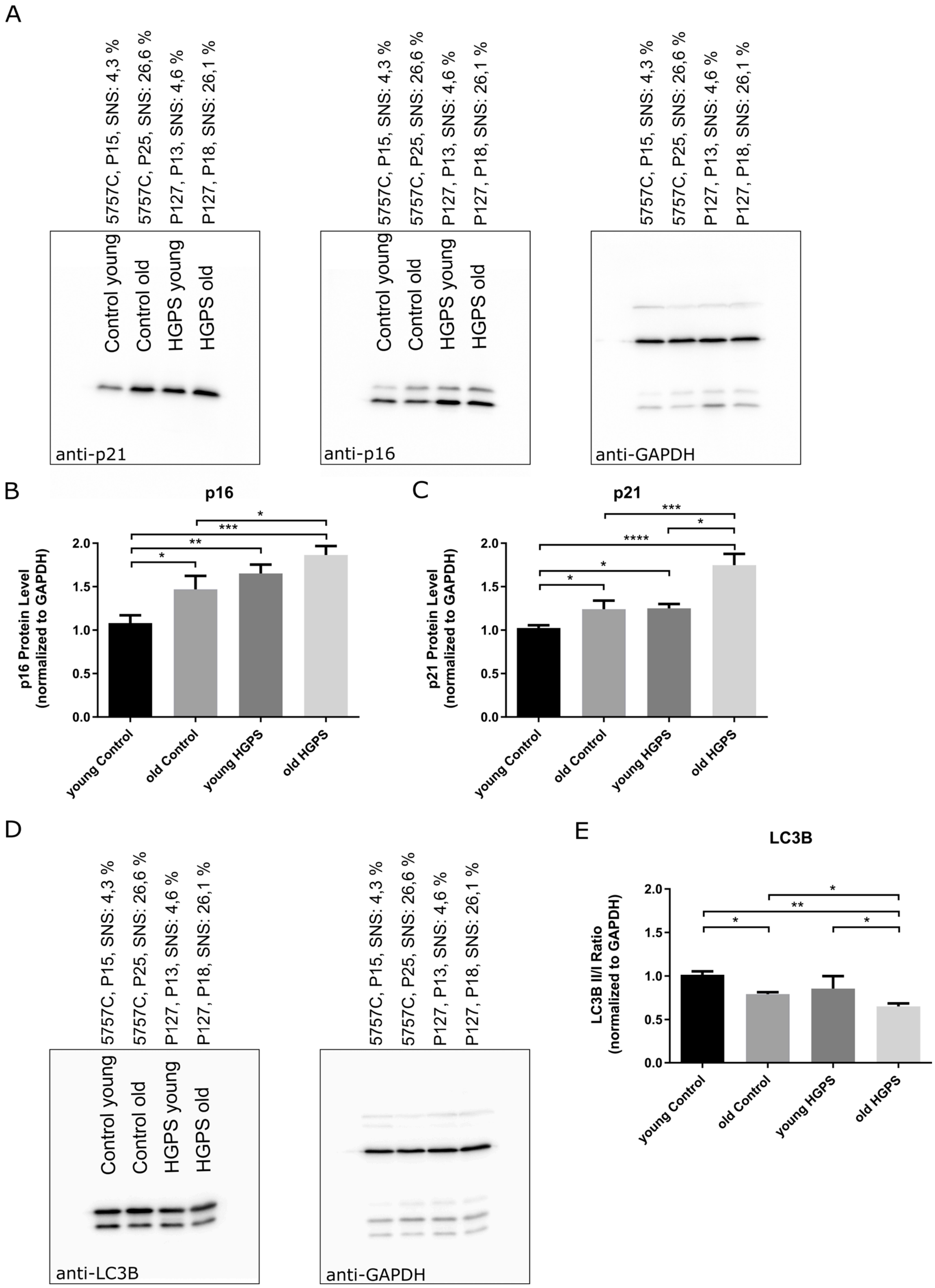

Figure S7: Western Blot Analysis of young and old Control and HGPS Fibroblasts without treatment**.** Control fibroblasts (5757C, F369, M368) and HGPS fibroblasts (P003, P127, P271) with a senescence level of approx. 5% (young) and a senescence level over 25% (old) were cultured for 7 days. (A) Representative images of western blot analyses for p16 and p21 protein levels in total protein extracts. Normalized to GAPDH. (B, C) Quantification of p16 and p21 protein level normalized to GAPDH (n=3). (D) Representative images of western blot analyses for LC3B protein levels in total protein extracts. Normalized to GAPDH. (E) Ratio of LC3B-II to LC3B-I (n=3). Graphs present mean ± SD; significance indicated by * $p < 0.05$; ** $p < 0.01$; *** $p < 0.001$; **** $p < 0.0001$, using an unpaired t-test and one-way anova. Panel A and D show full-length scans of western blots representative for panel B, C and E.

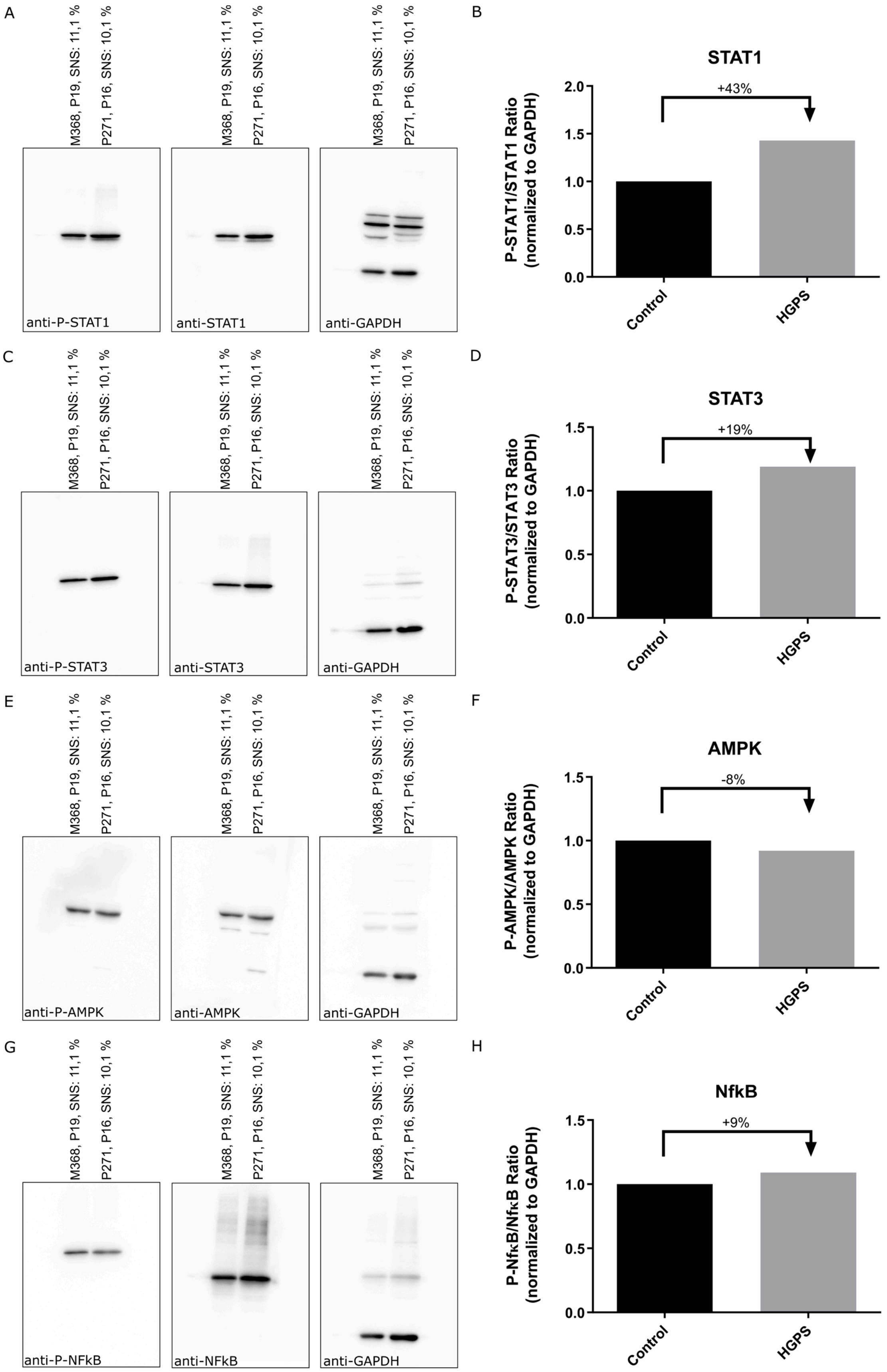

Figure S8: Western Blot Analysis of mock treated Control and HGPS Fibroblasts**.** Control fibroblasts (M368, P19) and HGPS fibroblasts (P271, P16) with a senescence level of approximately 10-15% were treated for 7 days with no compound (mock). Panels (A, C, E, G) show representative Western blot images for phosphorylated and total forms of STAT1 (A), STAT3 (C), AMPK (E), and NFκB

(G). Panels (B, D, F, H) depict the ratios of phosphorylated to total STAT1 (B), STAT3 (D), AMPK (F), and NFκB (H) in both control and HGPS fibroblasts without treatment. Graphs present mean (n=1); significance indicated by * $p < 0.05$; ** $p < 0.01$; *** $p < 0.001$; **** $p < 0.0001$, using an unpaired t-test. Panel A, C, E, and G show full-length scans of western blots.

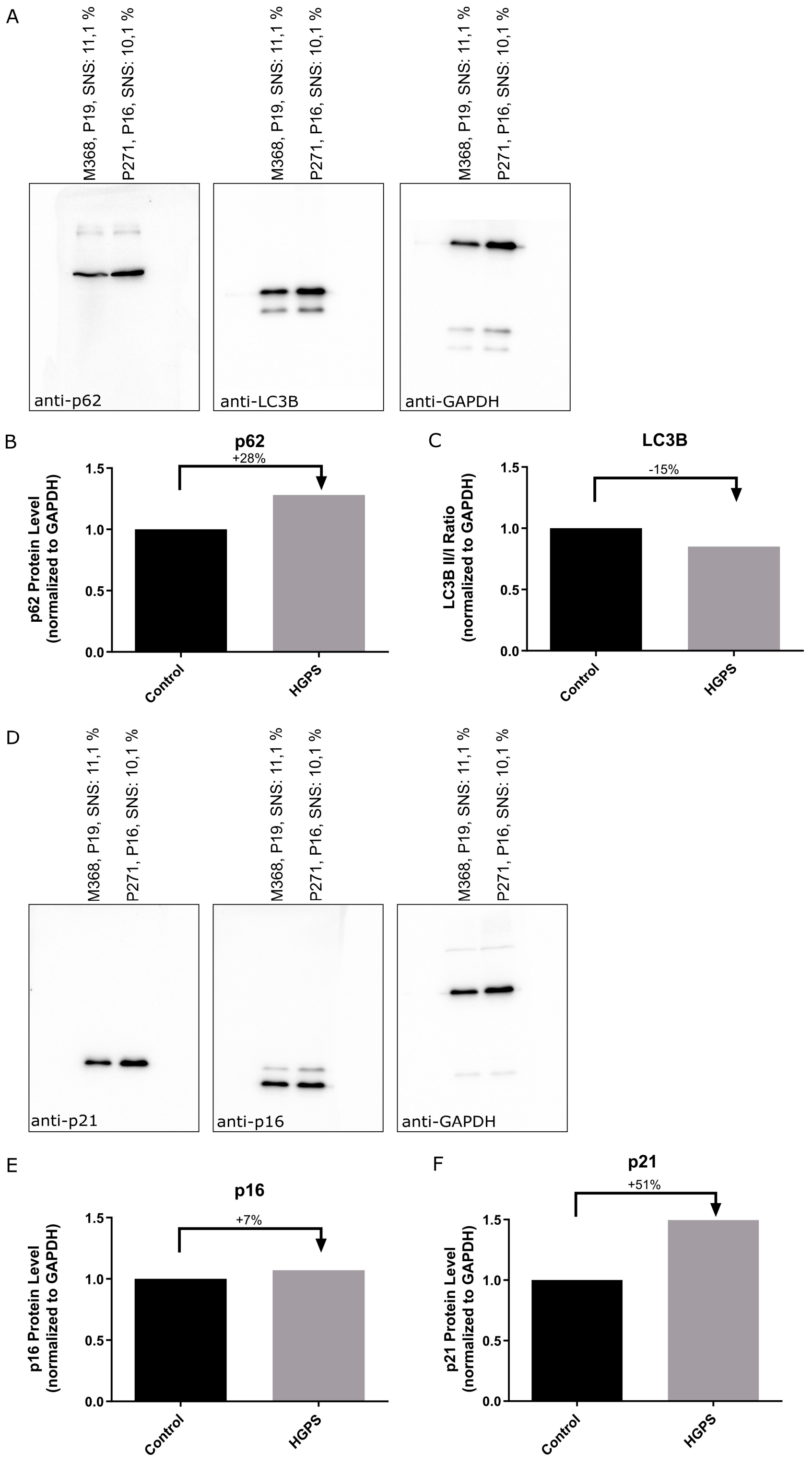

Figure S9: Western Blot Analysis of mock treated Control and HGPS Fibroblasts (A-E). Control fibroblasts (M368, P19) and HGPS fibroblasts (P271, P16) with a senescence level of approximately 10-15% were treated for 7 days with no compound (mock). Representative Western blot images for p62 (A), LC3B (A), p16 (D) and p21 (D). (B, E, F) Quantification of p62, p16, p21 protein levels normalized to GAPDH. (C) Ratio of LC3B-II to LC3B-I. Graphs present mean (n=1); significance indicated by * $p < 0.05$; ** $p < 0.01$; *** $p < 0.001$; **** $p < 0.0001$, using an unpaired t-test. Panel J and M show full-length scans of western blots.

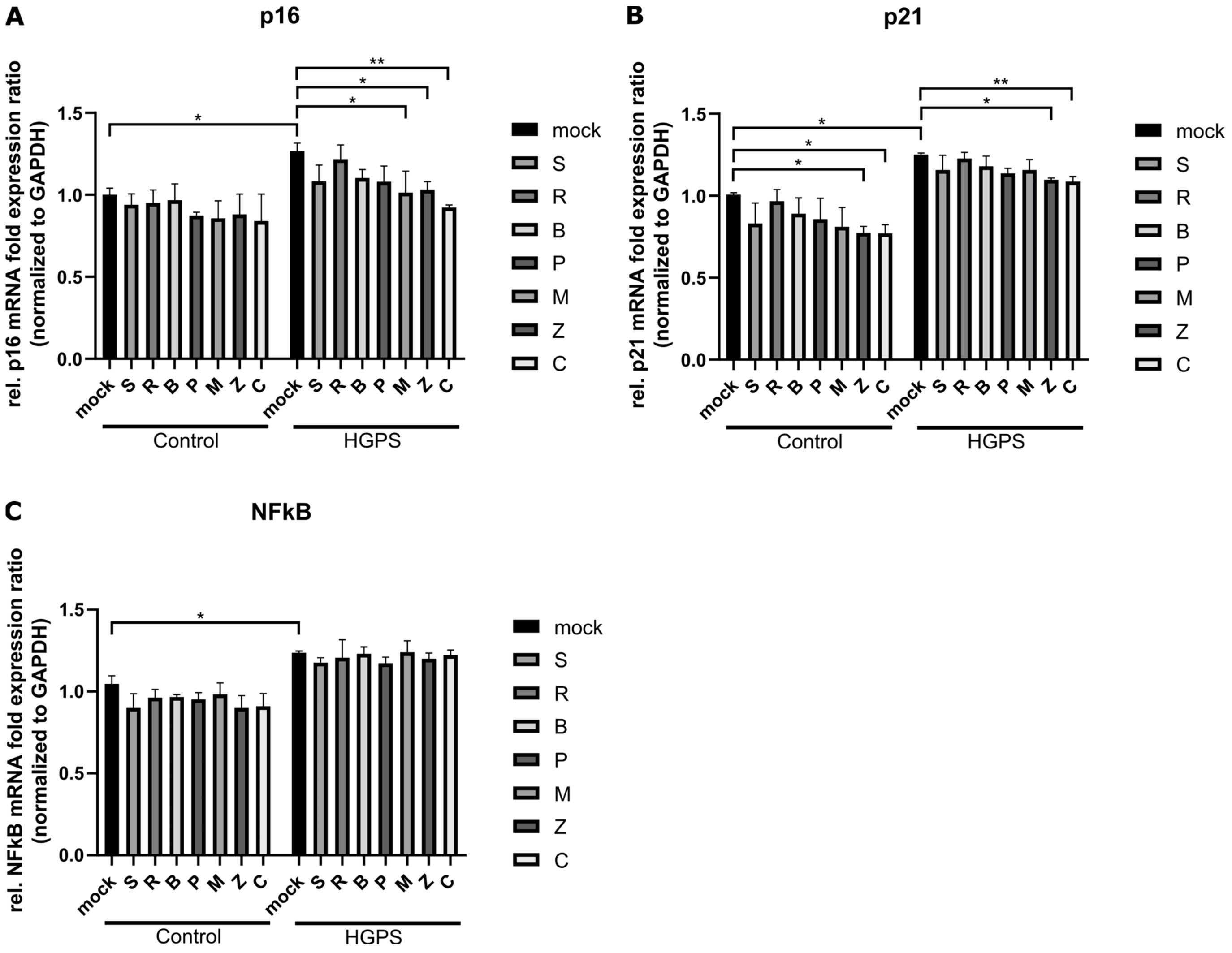

Figure S10: Replicative senescence levels profiles and NFκB expression of Control and HGPS fibroblasts under different compound treatment conditions. Control (5757C, F369, M368) and HGPS (P127, P164, P003) primary fibroblast cultures with a senescence level of approximately 15% were treated for 7 days with no compound (mock), 2.5 μM (+)-Pinoresinol (S), 10 μM Resveratrol (R), 2 μM Bisdemethoxycurcumin (B), 1 μM Pinosylvin (P), 1.5 μM Methyl P-Hydroxycinnamate (M), 2.5 μM cis-Pterostilbene (Z), and 10 μM (+)-Gallocatechin (C). Panel A-C despite the quantitative real-time PCR analysis of p16 (A), p21 (B) and NFκB (C). The relative expression ratio was normalized to expression of GAPDH. Graphs show mean ± SD (n = 3; * $p < 0.05$, ** $p < 0.01$), using two-way anova.

Table S1: Primers used for real-time quantitative PCR analysis

Primer sequence Target	Gene	Product size (bp)
FW: 5′- GGGGGCACCAGAGGCAGT -3′<br>REV: 5′- GGTTGTGGCGGGGGCAGTT -3′	p16^{INK4a}	150
FW: 5′- AGGTGGACCTGGAGACTCTCAG -3′<br>REV: 5′- TCCTCTTGGAGAAGATCAGCCG -3′	p21 (CDKN1A)	349
FW: 5′- GCAGCACTACTTCTTGACCACC -3′<br>REV: 5′- TCTGCTCCTGAGCATTGACGTC -3′	NfkB1	125
FW: 5′- GTCTCCTCTGACTTCAACAGCG -3′<br>REV: 5′- ACCACCCTGTTGCTGTAGCCAA -3′	GAPDH	51
